# The missense mutation C667F in murine β-dystroglycan causes embryonic lethality, myopathy and blood-brain barrier destabilization

**DOI:** 10.1242/dmm.050594

**Published:** 2024-06-18

**Authors:** Rui Lois Tan, Francesca Sciandra, Wolfgang Hübner, Manuela Bozzi, Jens Reimann, Susanne Schoch, Andrea Brancaccio, Sandra Blaess

**Affiliations:** ^1^ Neurodevelopmental Genetics, Institute of Reconstructive Neurobiology, Medical Faculty, University of Bonn, 53127 Bonn, Germany; ^2^ Institute of Chemical Sciences and Technologies ‘Giulio Natta’ (SCITEC)-CNR, 00168 Rome, Italy; ^3^ Biomolecular Photonics, Faculty of Physics, Bielefeld University, 33615 Bielefeld, Germany; ^4^ Dipartimento di Scienze Biotecnologiche di Base, Cliniche Intensivologiche e Perioperatorie. Sezione di Biochimica. Università Cattolica del Sacro Cuore di Roma, 00168 Rome, Italy; ^5^ Department of Neurology, Neuromuscular Diseases Section, University Hospital Bonn, 53127 Bonn, Germany; ^6^ Synaptic Neuroscience Team, Institute of Neuropathology, Medical Faculty, University of Bonn, 53127 Bonn, Germany; ^7^ School of Biochemistry, University Walk, University of Bristol, Bristol BS8 1TD, UK

**Keywords:** Aquaporin 4, Blood-brain barrier, Dystroglycan, Dystroglycanopathies, Missense mutation, Myopathy

## Abstract

Dystroglycan (DG) is an extracellular matrix receptor consisting of an α- and a β-DG subunit encoded by the *DAG1* gene. The homozygous mutation (c.2006G>T, p.Cys669Phe) in β-DG causes muscle-eye-brain disease with multicystic leukodystrophy in humans. In a mouse model of this primary dystroglycanopathy, approximately two-thirds of homozygous embryos fail to develop to term. Mutant mice that are born undergo a normal postnatal development but show a late-onset myopathy with partially penetrant histopathological changes and an impaired performance on an activity wheel. Their brains and eyes are structurally normal, but the localization of mutant β-DG is altered in the glial perivascular end-feet, resulting in a perturbed protein composition of the blood-brain and blood-retina barrier. In addition, α- and β-DG protein levels are significantly reduced in muscle and brain of mutant mice. Owing to the partially penetrant developmental phenotype of the C669F β-DG mice, they represent a novel and highly valuable mouse model with which to study the molecular effects of β-DG functional alterations both during embryogenesis and in mature muscle, brain and eye, and to gain insight into the pathogenesis of primary dystroglycanopathies.

## Teaser

**Summary:** The characterization of a mouse model of a homozygous dystroglycan mutation causing muscle-eye-brain disease reveals defective embryonic development, myopathy and a disrupted gliovascular unit, offering novel insights into dystroglycanopathy pathogenesis.

**Research Simplified**

Muscle-eye-brain (MEB) disease is a rare disease that is characterised by progressive muscle degeneration, severe defects in brain anatomy, eye malformations and developmental delay. A mutation in the gene that codes for dystroglycan – a protein complex with essential roles in skeletal muscles, the brain and the eye – has been implicated in causing MEB disease. Understanding how this specific mutation causes MEB disease can help researchers develop better diagnostics for and potential therapeutics against this deadly disease.

The authors introduced the MEB disease-associated mutation in the dystroglycan-coding gene in mice. Although most of the mice carrying this mutation die during embryonic development, the few mutant mice that survived were mostly healthy during the first year of their lives. However, their muscle fibres showed severely reduced dystroglycan protein levels and adult mice exhibited myopathy (dysfunction of muscle fibres). One-year-old mutant mice also displayed muscle-specific reduced running speed, without any impairment in motivation or motor coordination. Finally, the authors found that the dystroglycan mutation disrupted function of the protein complex that is critical for the organisation and stability of the blood-brain barrier.

This study recapitulated some of the detrimental symptoms of human MEB disease in a laboratory mouse model and showed that the MEB disease-associated mutation in dystroglycan disrupts critical brain functions. As humans and mice share several common physiological features, further research can help develop therapeutics for MEB disease in humans.

## INTRODUCTION

The dystroglycan (DG) protein complex is central in several physiological and pathological contexts, playing a particularly important role in skeletal muscle, brain and eye (Adams and Brancaccio, 2015). It is composed of two subunits: the extracellular and highly glycosylated α-DG, and the transmembrane β-DG, which act as a molecular link forming an axis between the extracellular matrix (ECM) and the cytoskeleton (Ervasti and Campbell, 1993; Ibraghimov-Beskrovnaya et al., 1992). The β-DG subunit establishes contacts with dystrophin and the cytoskeleton. DG is the major non-integrin cell-ECM adhesion complex and provides stability to various tissues such as skeletal and smooth muscle, and the central and peripheral nervous systems. In addition, DG is involved in the stabilization of cell-matrix interfaces, such as those at the neuromuscular junction, at the interface between endothelial cells and astrocytic end-feet at the blood-brain barrier (BBB), at podocyte-glomeruli basement membrane contacts and at the epithelia-connective tissue border in the lung (Barresi and Campbell, 2006; Bozzi et al., 2009; Winder, 2001). Furthermore, DG plays a very early and crucial role during mouse embryogenesis due to the essential role of DG in maintaining Reichert's membrane, which is a specialized basement membrane involved in rodent embryonic development (Williamson et al., 1997). Mutations that disrupt the function of human DG, its associated proteins or enzymes important for its post-translational maturation lead to various forms of muscular dystrophy, which can be accompanied by distinctive eye and brain phenotypes (Ackroyd et al., 2008; Bianchini et al., 2014; Liu et al., 2006).

Most of the DG-related muscular dystrophies result from alterations of the α-DG glycosylation shell observed in secondary dystroglycanopathies that are due to genetic abnormalities of glycosyltransferases involved in the glycosylation of α-DG (Endo, 2014; Fortunato et al., 2014; Muntoni et al., 2008). The N-terminal domain of α-DG (Bozic et al., 2004; Brancaccio et al., 1997) may also be involved, as it controls the binding of DG to glycosyltransferases or to additional factors that are important for the post-translational maturation of DG in the Golgi (Kanagawa et al., 2004). However, the exact relationship between the level of α-DG glycosylation and its pathophysiological consequences remains to be elucidated (Jimenez-Mallebrera et al., 2008). Although an increasing number of severe neuromuscular diseases resulting from *DAG1* mutations, the so-called primary dystroglycanopathies, have been identified (Dai et al., 2019; Dong et al., 2015; Geis et al., 2013; Hara et al., 2011; Özyilmaz et al., 2019), the underlying molecular basis of these diseases remains largely unknown (Brancaccio, 2019).

One of these primary dystroglycanopathies results in muscle-eye-brain (MEB) disease with multicystic leukodystrophy, a severe neuromuscular condition with brain and eye abnormalities. It arises from a homozygous missense mutation (c.2006G>T) resulting in an amino acid substitution (p.Cys669Phe) in the ectodomain of β-DG and has been described in two human patients (i.e. two sisters, aged 2 and 3 years) (Geis et al., 2013). Beyond the identification of the initial carrier family and the characterization of histomorphological features (analyzed at an early postnatal stage and with no follow-up available), our previous work in transfected cell lines showed that the β-DG C667F mutation in mice (murine topological counterpart to the human C669F mutation) leads to a defective trafficking process of the entire adhesion complex, such that the complex remains mostly engulfed in the endoplasmic reticulum (Signorino et al., 2018). No further biochemical or molecular data or follow-up of the two patients are available for this primary dystroglycanopathy, and it remains unclear how the mutation in β-DG leads to the destabilization of the whole DG complex (DGC) and to the severe symptoms observed in human patients.

To unravel the underlying mechanisms of the pathology in muscle, brain and eyes, we generated and characterized a mouse model carrying the C667F mutation (corresponding to the C669F mutation in humans) within the ectodomain of β-DG. Our analysis shows that the mouse model of the C669F β-DG-associated MEB disease results in developmental delay and embryonic lethality in most mutant embryos. Mutant mice that develop to term reach late adulthood and show highly specific deficits in the glia-vascular unit in the central nervous system (CNS) as well as late-onset histopathological changes in skeletal muscle. Thus, our mouse model represents a novel and highly valuable tool with which to understand the mechanisms underlying various aspects of primary dystroglycanopathy pathology and to study the impact of β-DG dysfunction at the molecular level.

## RESULTS

### Generation and characterization of the *Dag1^C66F7/C667F^* mouse line

**Fig. 1.**
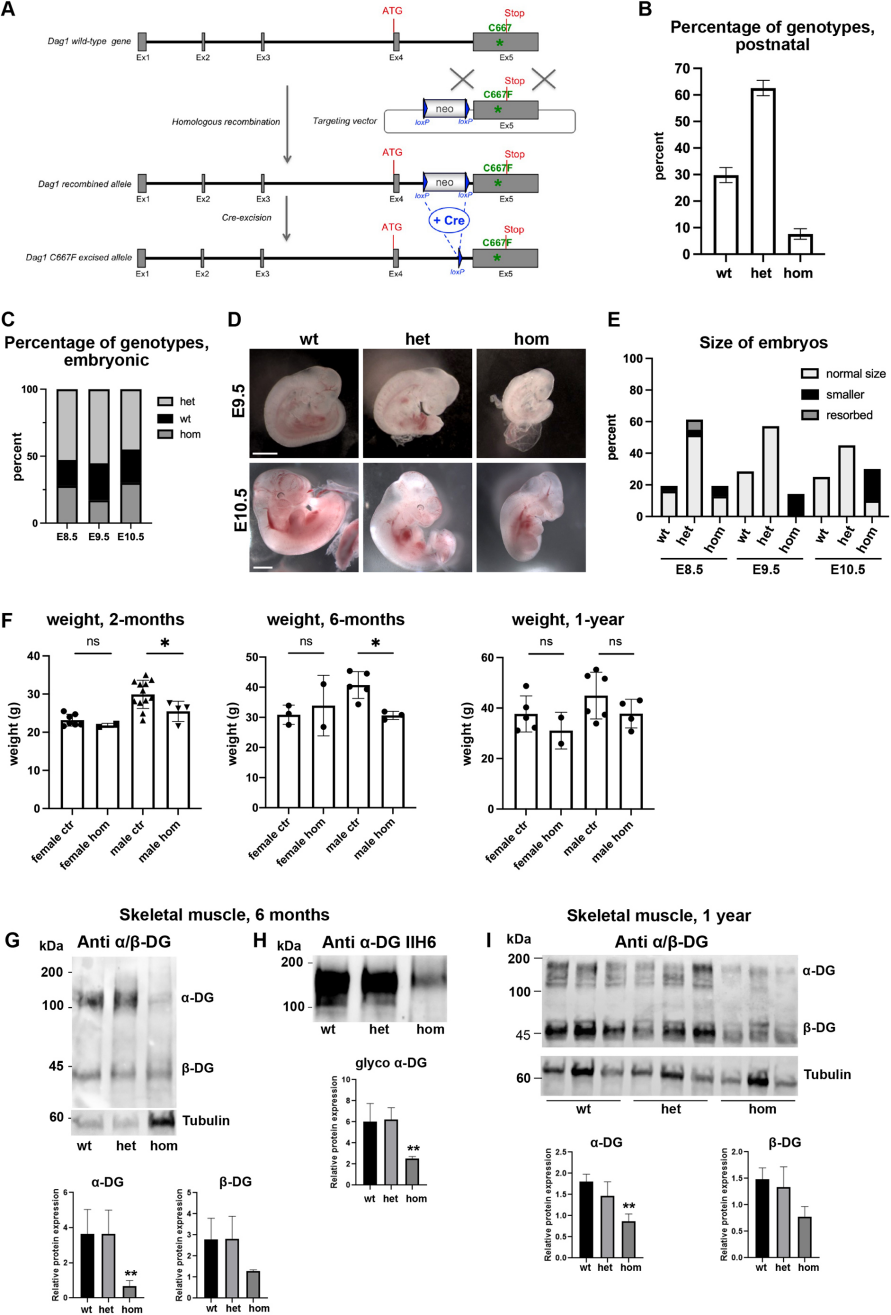
**Reduced DG protein levels in skeletal muscle and brain tissue of *Dag1^C667F/C667F^* mice.** (A-F) Generation and characterization of the *Dag1^C667/C667^* mouse line. (A) Strategy for the development of the *Dag1^C667^* mouse model. Shown are the wild-type allele, the targeting vector, the recombined locus and the point mutation allele after *in vivo* excision of the neo cassette. (B) *Dag1^C667F/C667F^* mice (hom) are not born at the expected Mendelian frequency of 25% (47 litters, 361 mice). (C) *Dag1^C667F/C667F^* are found with approximately the expected Mendelian frequency between E8.5 and E10.5. Number of analyzed embryos: 36 (E8.5), 47 (E9.5) and 20 (E10.5). (D) Whole-mount images of wild-type, heterozygous (het) and hom embryos at E9.5 and E10.5. Hom embryos are smaller than wild-type and het embryos. Scale bars: 1 mm. (E) Size of embryos between E8.5 and E10.5 for the different genotypes. Number of analyzed embryos: 36 (E8.5), 47 (E9.5) and 20 (E10.5). (F) Hom male mice weigh significantly less than control (ctr) mice (genotype: *Dag1^C667F/+^* or *Dag1^+/+^*) at 2 and 6 months of age, but not at 1 year of age. There is no significant difference between female control and mutant mice at the ages analyzed. (G-I) Expression of DG was evaluated by western blot performed on skeletal muscle tissues (hindlimb hip adductor and abductor, and thigh knee flexor complexes) from 6-month- and 1-year-old wild-type, het and hom mice. Quantification of protein bands is reported as an average (6-month-old mice, *n*=4 for each genotype; 1-year-old mice, *n*=3 for each genotype) and presented as DG/tubulin ratio. (G,I) α- and β-DG subunits were analyzed in WGA enrichments using a polyclonal α/β-DG antibody recognizing both core proteins. (H) Glycosylated α-DG was detected using IIH6 monoclonal antibody. Data are mean±s.e.m. in B,F and ±s.d. in G-I. Statistical analysis was carried out using one-way ANOVA with Sidak's multiple comparison. *P<0.05; **P<0.01 (comparison of hom versus wild-type for G-I).

To generate a mouse line carrying the C667F mutation (corresponding to the C669F mutation in humans) in the ectodomain of β-DG, a point mutation was introduced into exon 5 of *Dag1* (Fig. 1A, see Materials and Methods for details). Mice heterozygous for the mutation (*Dag1^C667F/+^*) are viable, healthy and fertile. Homozygous (*Dag1^C667F/C667F^*) mice are not born with the expected Mendelian frequency (on average only 7.63±1.98% of mice per litter were homozygous), suggesting an embryonic phenotype that results in prenatal lethality but is only partially penetrant (Fig. 1B). As DG is essential for the maintenance of Reichert's membrane and thus embryonic development of mouse embryos (Williamson et al., 1997), we investigated at which stage embryonic development is impaired. Analysis at embryonic day (E) 8.5, E9.5 and E10.5 showed that homozygous embryos are present at almost the expected Mendelian frequency (average±s.d.: 19.34±10.87%; 12 litters analyzed; Fig. 1C) with an undisrupted embryonic morphology (Fig. 1D). However, the majority of the homozygous embryos was smaller than control littermates at E9.5 and E10.5 (Fig. 1D,E). To assess whether alterations in Reichert's membrane contribute to this embryonic phenotype in the *Dag1^C667F/C667F^* embryos, we performed immunostaining for laminin and for DG (with an antibody recognizing the core protein of both α- and β-DG) at E8.5. This showed that in most homozygous embryos, laminin was irregularly deposited in Reichert's membrane and DG was not or was only weakly expressed at the interface between the parietal endoderm, Reichert's membrane and the trophectoderm (Fig. S1A,B).

Despite this severe embryonic phenotype, *Dag1^C667F/C667F^* mice that develop to term survive to adulthood, are healthy and have no apparent behavioral phenotype in the home cage until over 1 year of age. Furthermore, homozygous mutants appear to be fertile (two matings resulted in two litters of two and four pups, respectively). However, comparison with littermates showed that *Dag1^C667F/C667F^* males weigh significantly less than control littermates at 2 and 6 months of age (Fig. 1F). As the human patients were affected by MEB disease, we next focused our analysis on the approximately one-third of the mutant mice that were born to study the effect of the C667F mutation on muscle, brain and eye development and maintenance.

### DG protein levels are reduced in skeletal muscle and brain tissue of *Dag1^C667F/C667F^* mice

First, we investigated whether the mutation leads to alterations in DG protein expression and processing in muscle and brain (Fig. 1G-I; Fig. S1C-G). Based on our previous observations in transfected cell lines (Signorino et al., 2018), we expected an alteration in α/β-DG processing or trafficking and thus a potential reduction in protein levels in homozygous mice. Lysates and succinylated wheat germ agglutinin (WGA) enrichments from brain and muscle tissues were subjected to a western blot analysis with an antibody recognizing the core protein of both α- and β-DG. Although DG was cleaved into its α- and β-subunits, α- and β-DG protein levels were substantially reduced in skeletal muscle and brain of *Dag1^C667F/C667F^* mice compared with wild-type or heterozygous controls (Fig. 1G,I; Fig. S1C). Western blot analysis with the anti-α-DG IIH6 antibody, which detects the glycan moiety responsible for α-DG binding to laminin and is commonly used to identify glycosylated α-DG, showed that α-DG could be detected in muscle and brain tissues from homozygous animals, indicating that α-DG is glycosylated in *Dag1^C667F/C667F^* mice (Fig. 1H; Fig. S1D). Thus, the C667F mutation results in reduced levels of DG but no alteration in its processing or glycosylation in the mouse model.

To investigate the mechanisms underlying the reduced DG levels, we performed quantitative RT-PCR of brain tissue to exclude that the decreased DG expression levels were due to reduced *Dag1* transcription. This analysis revealed no significant difference in *Dag1* mRNA levels between homozygous, heterozygous and wild-type animals (Fig. S1E). In addition, analysis of polyubiquitylation levels of total protein extracts from brain and muscle samples excluded hyperactivation of the proteasome-ubiquitin protein degradation pathway (Fig. S1F). To examine whether the reduced level of α-DG affects signaling pathways downstream of DG-mediated cell-ECM interaction, we analyzed the pI3K/AKT pathway and found no difference in the pAKT/AKT ratio between control and *Dag1^C667F/C667F^* mice (Fig. S1G).

Given the reduced DG expression in muscle and brain tissue from adult *Dag1^C667F/C667F^* mice, we next investigated the effect of the mutation on DG protein localization in muscle fibers and on skeletal muscle integrity and function. We then conducted a detailed analysis of DG localization and potential phenotypes in the brain and retina of the mutant mice.

### α- and β-DG are localized at the sarcolemma in the skeletal muscle of *Dag1^C667F/C667F^* mice

**Fig. 2.**
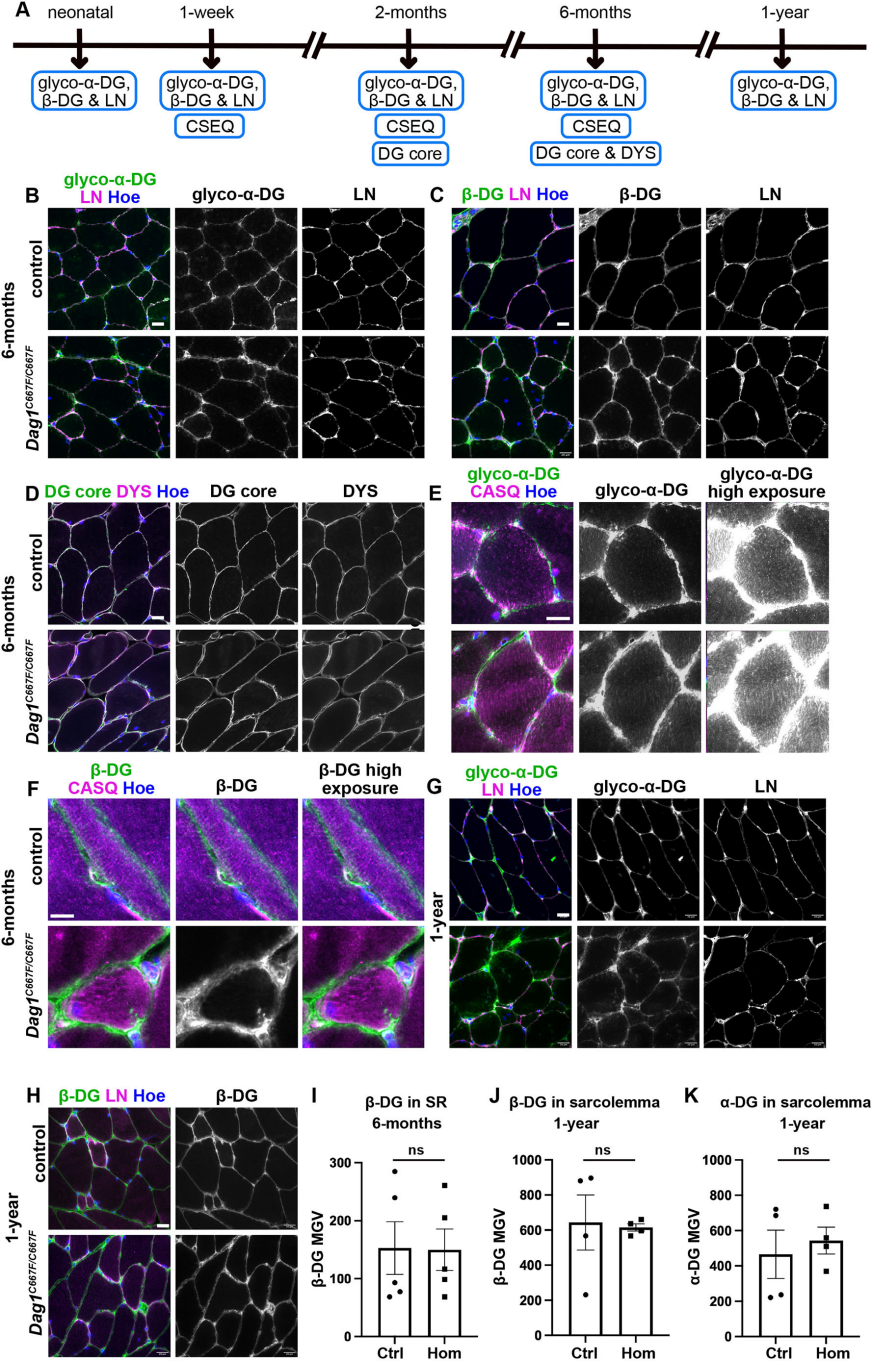
**α- and β-DG are localized at the sarcolemma in skeletal muscle of *Dag1^C667F/C667F^* mice.** (A) Timeline summarizing which experiments were performed at which postnatal stages. (B-H) Cross-section through the quadriceps femoris muscle of 6-month- or 1-year-old mice. (B,C) Immunostaining for glycosylated (glyco) α-DG or β-DG in combination with laminin (LN) and Hoechst (Hoe) in 6-month-old control and *Dag1^C667F/C667F^* mice. (D) Immunostaining for the DG core protein (α/β-DG) and dystrophin (DYS). (E,F) Immunostaining for glyco α-DG or β-DG in combination with the sarcoplasmic reticulum (SR) marker calsequestrin (CASQ). The middle and the right panels show the same area with different adjustments in the intensity levels to visualize the signal in the membrane (middle panel) and the weak signal for glyco α- or β-DG in the SR (right panel). (G,H) Immunostaining for glyco α- or β-DG in combination with laminin (LN) and Hoechst (Hoe) in 1-year-old control and *Dag1^C667F/C667F^* mice. Scale bars: 20 μm in B-D,G,H; 10 μm in E,F. (I-K) Quantification of the mean gray value (MGV) of β-DG in the SR (CASQ positive area) of 6-month-old mice (*n*=5 mice per group) (I), MGV of β-DG (J) or glyco α-DG (K) at the sarcolemma of 1-year-old mice (*n*=4 mice per group). Data are mean±s.e.m. Statistical analysis was carried out using a two-tailed unpaired *t*-test. ns, not significant.

In muscle fibers, α- and β-DG are localized at the sarcolemma and α-DG binds to its ECM partner, laminin. To determine whether the C667F mutation alters the localization of DG in muscle fibers, we examined whether α- and β-DG could be detected at the sarcolemma in *Dag1^C667F/C667F^* mice (Fig. 2A-K). Sections of hindlimb muscle (tibialis, biceps and triceps) were immunostained for glycosylated α-DG, β-DG or α/β-DG core protein in conjunction with laminin or dystrophin (DYS) at multiple time points (Fig. 2B-D,G,H). For these and all subsequent experiments, both heterozygous and wild-type mice were used as controls, as DG levels were not reduced in the heterozygous mice (Fig. 1; Fig. S1). Immunostaining revealed that α- and β-DG were present at the sarcolemma in homozygous mice and expression was not obviously reduced compared with that in controls at any of the time points analyzed (Fig. 2B-D,G,H,J,K). Localization of DYS in the sarcolemma and laminin around the individual muscle fibers was also similar in mutant and control mice (Fig. 2B-D,G,H). As the C667F mutant protein accumulates in the ER of transfected cell lines (Signorino et al., 2018), we investigated whether α- and β-DG protein are partially retained in the sarcoplasmic reticulum (SR). Therefore, we performed double staining for calsequestrin (CASQ), a marker for the SR, and for either glycosylated α-DG or β-DG in muscle from 1-week- and 6-month-old mice. We could not detect any obvious increase in α- or β-DG levels in the SR of the *Dag1^C667F/C667F^* mice at these stages (Fig. 2E,F,I). These data indicate that the mutation in the ectodomain of β-DG does not result in a severe disruption of DG processing or subcellular localization in skeletal muscle but rather in an overall reduction in DG protein levels evident only in western blot analysis.

### A late-onset histopathological phenotype is observed in skeletal muscle in a subset of *Dag1^C667F/C667F^* mice

**Fig. 3.**
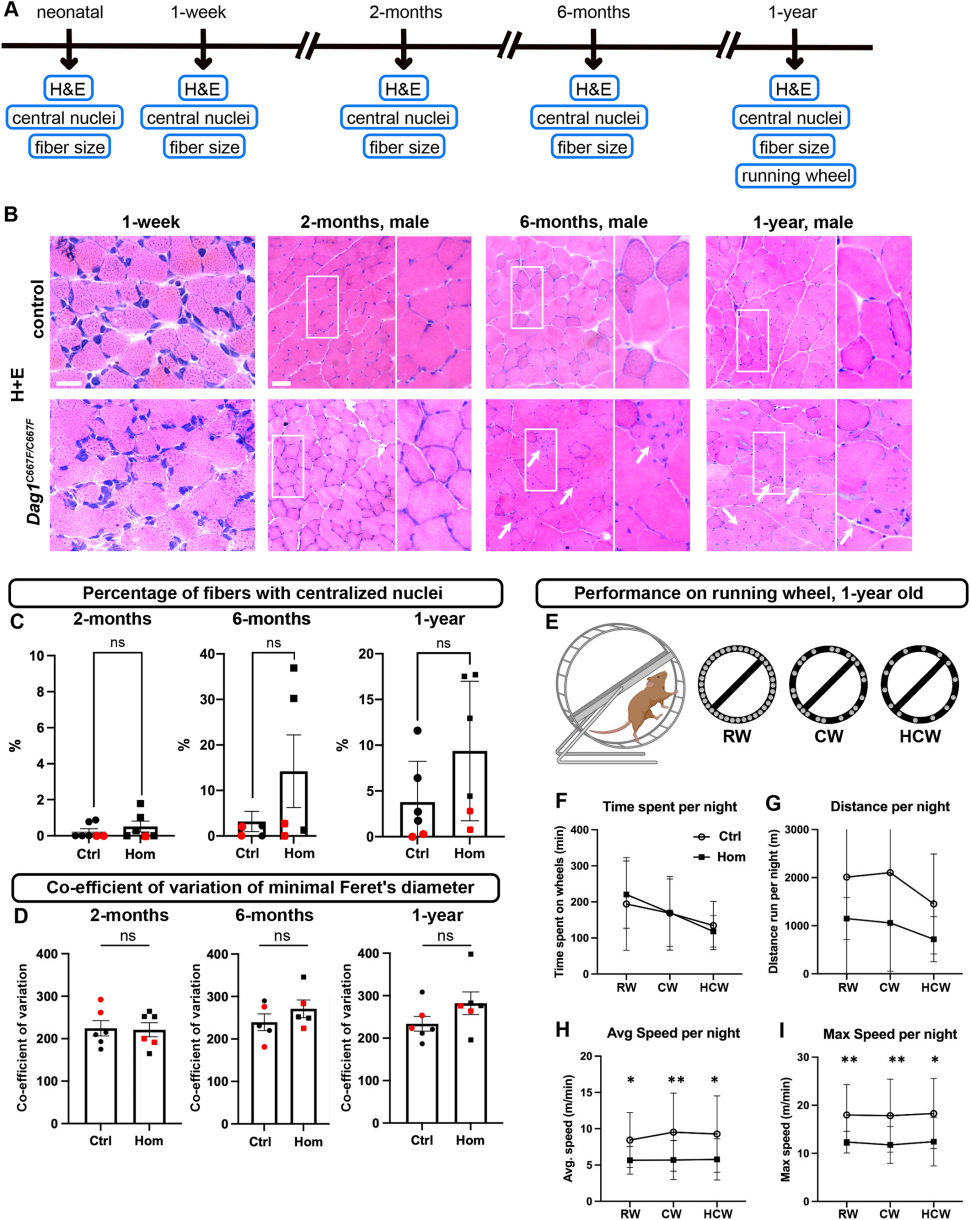
**A late-onset pathological phenotype in skeletal muscle in *Dag1^C667F/C667F^* mice.** (A) Timeline summarizing which experiments were performed at which postnatal stages. (B) Hematoxylin and Eosin (H&E) staining of cross-sections through the quadriceps femoris muscle at the indicated time points. 2-month-, 6-month- and 1-year-old muscle fibers: right panels show higher magnifications of the outlined areas in the left panels. Arrows indicate the presence of centralized nuclei in 6-month- and 1-year-old muscle fibers. Scale bars: 20 μm for 1-week-old; 100 μm for 2-month- to 1-year-old. (C) Percentage of fibers with centralized nuclei. Although overall there is no significant difference between control (Ctrl) and *Dag1^C667F/C667F^* (Hom) mice, there are several male Hom mice (black squares) with a high percentage of fibers with centralized nuclei. (D) The variance coefficient (VC) of Feret's diameter is not significantly different between Ctrl and Hom mice at the stages analyzed. (C,D) Black circles or squares, male mice; red circles or squares, female mice. Data are mean±s.e.m. Statistical analysis was carried out using a two-tailed unpaired *t*-test with Welch's corrections (C, 2-month-old; D, 1-year-old), a Mann–Whitney test (C, 6-month- and 1-year-old) or a two-tailed unpaired *t*-test (D, 2- and 6-month-old). (E-I) 1-year-old mice were given access to an activity wheel in their home cage. (E) Mice had access to a 33-rung wheel (regular wheel, RW) for 2 days. Subsequently, mice had access to an irregularly spaced 22-rung wheel (complex wheel, CW) for 4 days, followed by access to an irregularly spaced 14-rung wheel (highly complex wheel, HCW) for 2 days. Created with Biorender.com. (F,G) Ctrl and Hom mice spent a similar amount of time per night on the RW, CW or HCW, and the distance covered per night was not significantly different between Ctrl and Hom mice. (H,I) Average speed and maximum speed per night are significantly lower in Hom mice compared with Ctrl mice on the RW, CW and HCW. *n*=6 mice per group. Data are mean±s.e.m. Statistical analysis was carried out using two-way ANOVA with Sidak's multiple comparison. *P<0.05; **P<0.01.

Given the reduction in α- and β-DG protein levels in skeletal muscle of homozygous animals, we examined whether the hindlimb muscle (quadriceps femoris muscle) of homozygous mutant mice showed histopathological changes (Fig. 3A-D). Both heterozygous and wild-type mice were used as controls because the muscle histology of the heterozygous mice was indistinguishable from that of wild-type mice (data not shown). One sign of muscular dystrophy and the subsequent muscle fiber regeneration is the presence of nuclei in the center of the muscle fiber. Histological analysis of neonatal, 1-week- or 2-month-old mice did not show an overt phenotype (Fig. 3B,C). However, in 6-month- and 1-year-old mice, we found a substantial increase in the percentage of fibers with nuclei in the central region in two of the male homozygous mice (Fig. 3C). Next, we performed a quantitative analysis of the muscle fiber cross-sectional area (2- and 6-month-old mice) and minimum Feret's diameter (neonatal, 1-week-, 2-month-, 6-month- and 1-year-old mice) in homozygous and control mice, as the degree of variability in muscle fiber size may be an indicator of muscular dystrophy (Briguet et al., 2004). This analysis showed no significant difference in the coefficient of variation (VC) of either the cross-sectional area or of the minimum Feret's diameter of muscle fibers in homozygous compared with control mice at any stage analyzed (Fig. 3D). Even when these two measures were specifically compared between control males and males with an increased percentage of fibers with central nuclei, no significant difference was found (data not shown). Nevertheless, histograms of the minimum Feret's diameter of mice at 6 months and 1 year of age showed a broadening and flattening of the distribution in male and female *Dag1^C667F/C667F^* mice compared with controls (Fig. S2A,B). Taken together, these data suggest that the mutation in β-DG results in a subtle, late-onset and partially penetrant histopathological phenotype in the muscle of adult (older than 2 months) *Dag1^C667F/C667F^* mice.

Finally, we tested whether the severity of the histopathological phenotype correlated with the level of DG expression or localization at the sarcolemma or SR by blotting the percentage of central nuclei against either the level of DG protein expression detected by western blot or the intensity of DG staining in the sarcolemma or SR detected by immunostaining for individual mice. This analysis showed no obvious correlation between DG protein expression or DG localization in the sarcolemma and the histopathology. Interestingly, the two *Dag1^C667F/C667F^* mice with the highest percentage of central nuclei at 6 months of age also had the highest signal for β-DG in the SR. However, a clear conclusion regarding the correlation between β-DG localization in the muscle fiber and the histopathological phenotype cannot be drawn due to the small number of mutant mice analyzed (*n*=5) and the fact that two control mice with no histopathological phenotype also showed relatively high levels of β-DG in the SR (Fig. S3).

### Reduced running capacity in 1-year-old *Dag1^C667F/C667F^* mice

Given the reduced DG protein levels and the subtle histopathological phenotype in the hindlimb muscle of aged mice, we next investigated whether skeletal muscle function is altered in *Dag1^C667F/C667F^* mice. One-year-old control and *Dag1^C667F/C667F^* mice were individually housed in cages containing an activity wheel and their performance was automatically monitored (Novak et al., 2012). Voluntary wheel running has been used in several studies to assess endurance in mice with skeletal muscle dysfunction (Elbaz et al., 2019; Mosca et al., 2013; Ruiz et al., 2022). In our paradigm, we also tested for effects on movement coordination: the wheel was made more complex over time by removing individual rungs (Fig. 3E). Maximum and average running speed, total running time and distance for individual sessions or the entire dark period were compared between control and *Dag1^C667F/C667F^* mice for the three wheel types (Fig. 3F-I; Fig. S2C-H). On the regular wheel, the average and the maximum running speed per dark phase and per session were reduced in the mutants as compared with the controls, suggesting that their performance was impaired (Fig. 3H,I; Fig. S2F,G). The performance levels observed on the regular wheel were maintained on the more complex wheel types in both control and mutant animals, indicating that motor coordination was not impaired in the *Dag1^C667F/C667F^* mice (Fig. 3F-I; Fig. S2C-H). The total time spent on the wheel per dark period, the length and number of individual sessions were not significantly different between control and mutant mice, indicating that endurance and motivation to run were not altered in the mutants (Fig. 3F; Fig. S2C,D). Likely due to the decreased speed of the mutant mice compared with controls, the total running distance per dark period appeared to be reduced (without reaching significance) and the mean distance per session was significantly reduced for the complex and highly complex wheel (Fig. 3G; Fig. S2E). In conclusion, these data show that 1-year-old *Dag1^C667F/C667F^* mutant mice have a diminished running capacity, as manifested by reduced speed compared with control mice, whereas time spent on the wheel, motivation and coordination are not overtly affected in the homozygous mutant mice, suggesting a muscle-specific phenotype.

### No obvious anatomical changes in brain or eye of postnatal *Dag1^C667F/C667F^* mice

**Fig. 4.**
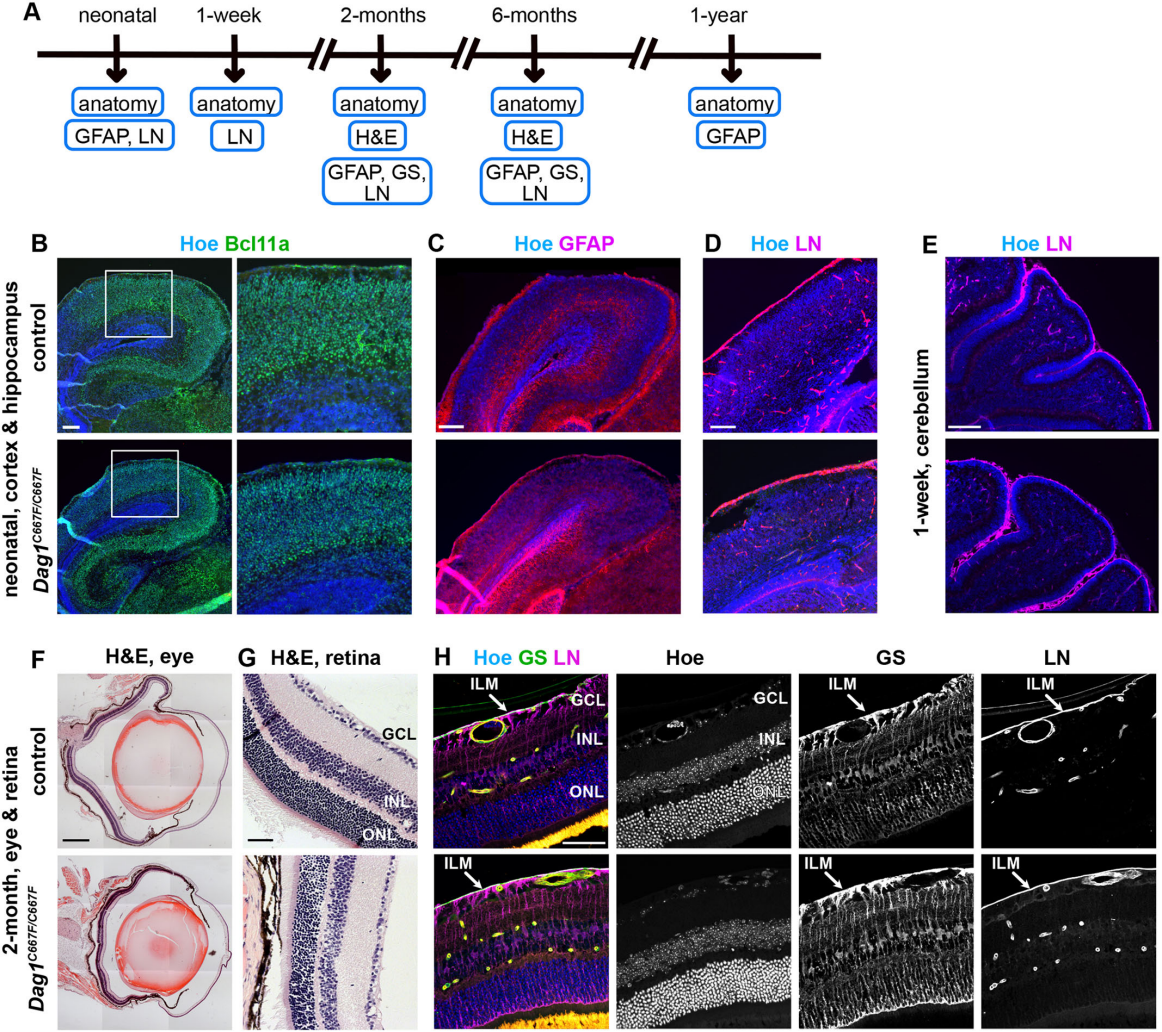
**Brain and eye show no obvious anatomical changes in *Dag1^C667F/C667F^* mice.** (A) Timeline summarizing which experiments were performed at which postnatal stages. (B-E) Analysis of brain anatomy in neonatal control and *Dag1^C667F/C667F^* mice. (B,C) Immunostaining for the cortical neuron marker BCL11A (B) or the astrocyte marker GFAP (C) in combination with Hoechst (Hoe). (D) Laminin (LN) is expressed around blood vessels and at the pial surface of the neonatal cortex in control and *Dag1^C667F/C667F^* mice. (E) Cerebellar folia and the pial basement membrane (LN positive) covering cerebellar fissures and folia are established in the mutant mice. (F-H) Analysis of eye and retina phenotype in 2-month-old control and *Dag1^C667F/C667F^* mice. (F,G) Hematoxylin and Eosin (H&E) staining of cross-sections through the eye (F) and retina (G). GCL, ganglion cell layer; INL, inner nuclear layer; ONL, outer nuclear layer. (H) Immunostaining for LN and glutamine synthetase (GS), a marker for Müller glia cells, in combination with Hoechst. The inner limiting membrane (ILM) is present in homozygous mutant mice, as indicated by the presence of LN and GS-positive end-feet of Müller glial cells at the ILM. Scale bars: 200 μm in B-E; 500 μm in F; 50 μm G,H.

Conditional inactivation of *Dag1* in the mouse brain results in a detachment of radial glia end-feet from the basement membrane at the pial surface of the brain and a collapse of radial glia fibers. This results in the disruption and partial loss of the basement membrane, in layer defects in the cerebral cortex and cerebellum, and in the fusion of the cerebellar folia (Moore et al., 2002). Thus, if the C667F mutation in β-DG affects DG expression levels, localization or function in the developing brain, the *Dag1^C667F/C667F^* mice may have anatomically altered cortical structures. However, analysis of the layers in the cerebral and cerebellar cortex using Hoechst or neuronal markers (BCL11A for cortical neurons) at different postnatal time points (Fig. 4A) did not reveal any changes in the organization of the cerebral and cerebellar cortex in the homozygous mice (Fig. 4B-E). Furthermore, the distribution of GFAP-positive astrocytes and the localization of laminin at the pial surface appeared to be normal in *Dag1^C667F/C667F^* mice (Fig. 4C-E).

In addition to severe anatomical brain abnormalities, MEB disease associated with the DG C669F mutation is characterized by defects in the visual system, such as congenital glaucoma, myopia, retinal atrophy and/or juvenile cataracts in the human patients (Geis et al., 2013). To investigate whether these phenotypes are recapitulated in *Dag1^C667F/C667F^* mice, we isolated eyes at different postnatal and adult stages (Fig. 4A). Histological analysis of the eyes did not reveal any overt phenotypes (Fig. 4F), except for one 2-month-old mouse with a cataract in one eye (one out of 12 eyes) and a thin and disorganized ganglion cell layer, a phenotype that is also typically observed in glaucoma (Munemasa and Kitaoka, 2013). In the developing retina, DG is required to maintain the structural integrity of the inner limiting membrane (ILM) (Clements et al., 2017). Loss of DG function results in ILM degeneration and deficits in migration and axon guidance, as well as altered anatomical arrangement of neurons and retinal thinning. Histological examination of the retina in the *Dag1^C667F/C667F^* mutants did not reveal any obvious changes in retinal organization (Fig. 4G,H). Immunostaining for glutamine synthetase (GS), a marker for Müller glia cells, which span almost the entire retina, showed that these cells were structurally normal. Finally, immunostaining for laminin showed that the ILM was properly established in the homozygous mice (Fig. 4H).

Taken together, these data indicate that the mutation in β-DG does not disrupt the function of DG in organizing basement membranes at the pial surface of the brain or in the ILM in the retina in *Dag1^C667F/C667F^* mice. In addition, radial glia and Müller glia cells are established and maintained, and the anatomical development of the mouse brain and the retina are not overtly affected in *Dag1^C667F/C667F^* mice.

### The molecular composition of the glia-vascular unit is impaired in the brain of *Dag1^C667F/C667F^* mice

**Fig. 5.**
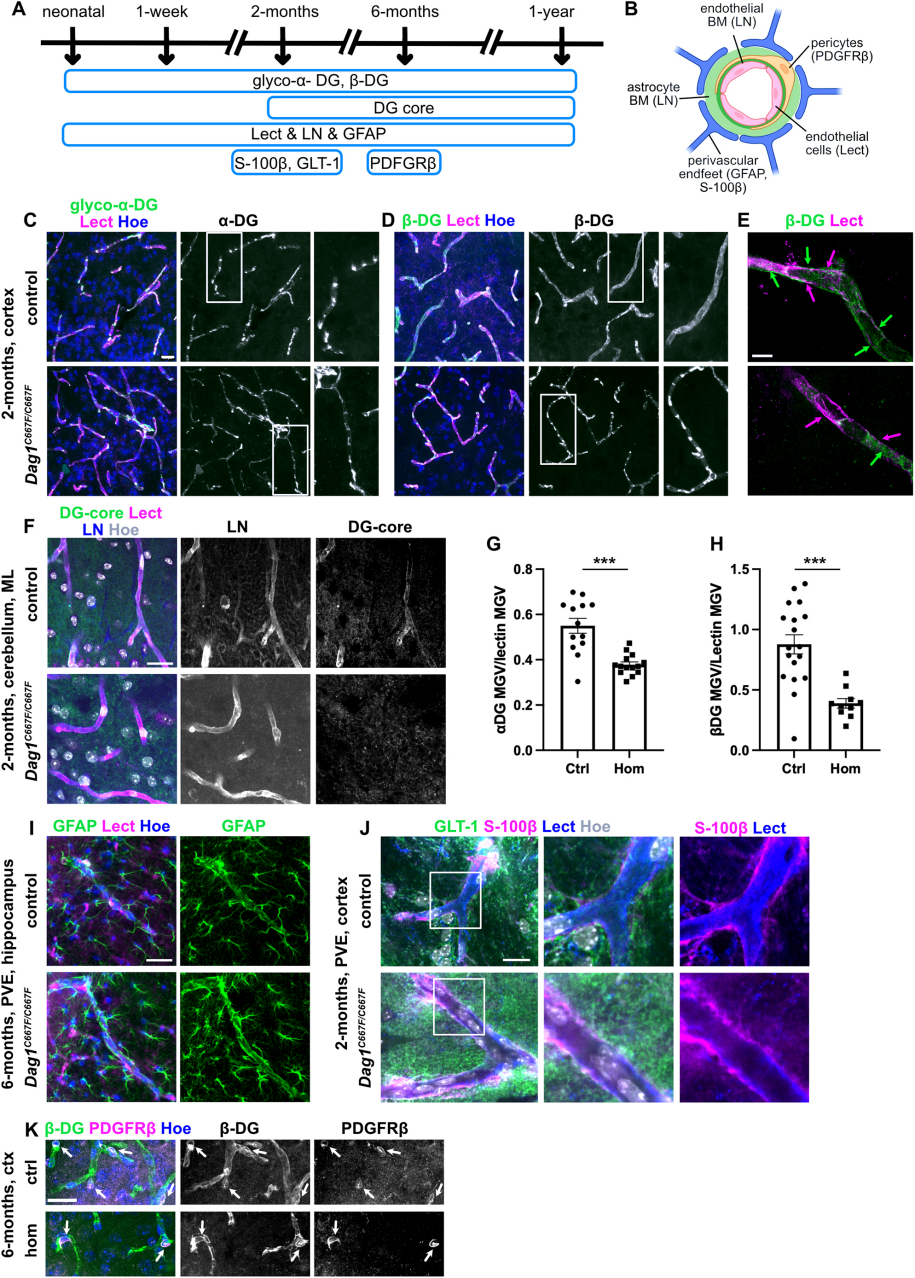
**Perivascular end-feet are formed in the brain of *Dag1^C667F/C667F^* mice.** (A) Timeline summarizing which experiments were performed at which postnatal stages. (B) Schematic representation of the cellular and ECM components of the BBB. Markers expressed in the different components are in brackets. BM, basement membrane; LN, laminin; Lect, lectin. Created with Biorender.com. (C-E) Immunostaining for glycosylated (glyco) α-DG or β-DG in combination with lectin (Lect) to visualize blood vessels and Hoechst (Hoe) in the cortex of 2-month-old mice. β-DG is abnormally clustered at blood vessels in *Dag1^C667F/C667F^* mice. (C,D) The right panels are higher magnifications of the areas outlined in the middle panels. (E) Super-resolution microscopy of single blood vessels (lectin-positive, magenta arrows) showing that β-DG (green arrows) is present in the perivascular space in control but not in *Dag1^C667F/C667F^* mice. 3 µm maximum intensity projections of *z*-stacks are shown. (F) Immunostaining for the α/β-DG core protein (DG-core) and laminin (LN) in combination with Lect and Hoe in the molecular layer of the cerebellum of 2-month-old mice. (G,H) Quantification of immunofluorescence intensity (mean gray value, MGV) of α-DG or β-DG expression in the PVE normalized to the MGV of lectin. *n*=5 mice per group; MGV was analyzed in three images per animal and included in the statistical analysis. Data are mean±s.e.m. Statistical analysis was carried out using a two-tailed unpaired *t*-test. ***P<0.001. (I) GFAP immunostaining in combination with Lect and Hoe visualizing astrocytes and their PVE surrounding a blood vessel in the hippocampus of 6-month-old control and *Dag1^C667F/C667F^* mice. (J) Immunostaining for GLT-1 to visualize astrocytes and S-100β, which localizes to PVE. (K) Immunostaining for β-DG and PDGFRβ to label pericytes. Pericytes (arrows) are located in the perivascular space and express β-DG in the cortex (ctx) of control (ctrl) and *Dag1^C667F/C667F^* (hom) mice. Scale bars: 20 μm in C,D,F,I,K; 5 μm in E; 10 μm in J.

Previously, complete inactivation of *Dag1* in the CNS was shown to alter astrocyte perivascular end-feet (PVE) and the BBB. PVE are highly specialized astrocytic processes that envelop the vasculature of the brain (Fig. 5). α- and β-DG are expressed in the PVE and also in endothelial cells (Fig. 5B) (Menezes et al., 2014; Zaccaria et al., 2001).

Immunostaining for glycosylated α-DG or β-DG combined with lectin to label blood vessels in the cerebral cortex showed that α- and β-DG were localized at blood vessels at the analyzed stages in control and mutant brains (Fig. 5A,C,D; Fig. S4A,B). However, the intensity of the fluorescent signal for either α-DG or β-DG at the PVE was reduced in 2-month-old *Dag1^C667F/C667F^* mice compared with controls (Fig. 5G,H), and β-DG was abnormally clustered around blood vessels in the brain of homozygous mice aged 2 months or older (Fig. 5D). Super-resolution microscopy of lectin and β-DG revealed that the perivascular localization of β-DG (surrounding the lectin staining on the extravascular site) was lost in homozygous animals (Fig. 5E; Movies 1 and 2). Furthermore, the DG core protein could be detected in the PVE in the molecular layer of the cerebellum in control mice, whereas it was absent in the PVE of *Dag1^C667F/C667F^* mice (Fig. 5F). Labeling for GFAP, GLT-1 (glutamate transporter 1, also known as EAAT1) and S-100β, three proteins expressed in astrocytes, two of which (GFAP and S-100β) are known to localize to the PVE, showed that the PVE are formed in *Dag1^C667F/C667F^* mice (Langer et al., 2017) (Fig. 5I,J; Fig. S4C). Laminin, a component of the basement membrane between blood vessels and the PVE, was preserved in the homozygous animals (Fig. 5F) as were the PDGFRβ-positive pericytes in the perivascular space (Fig. 5K) (Thomsen et al., 2017). Interestingly, co-immunostaining for β-DG and PDGFRβ showed that β-DG is expressed in pericytes and that this expression is maintained in the *Dag1^C667F/C667F^* brains. In conclusion, these data suggest that the reduced presence and clustering of DG along blood vessels is a consequence of DG mislocalization in the PVE rather than structural changes in the PVE.

**Fig. 6.**
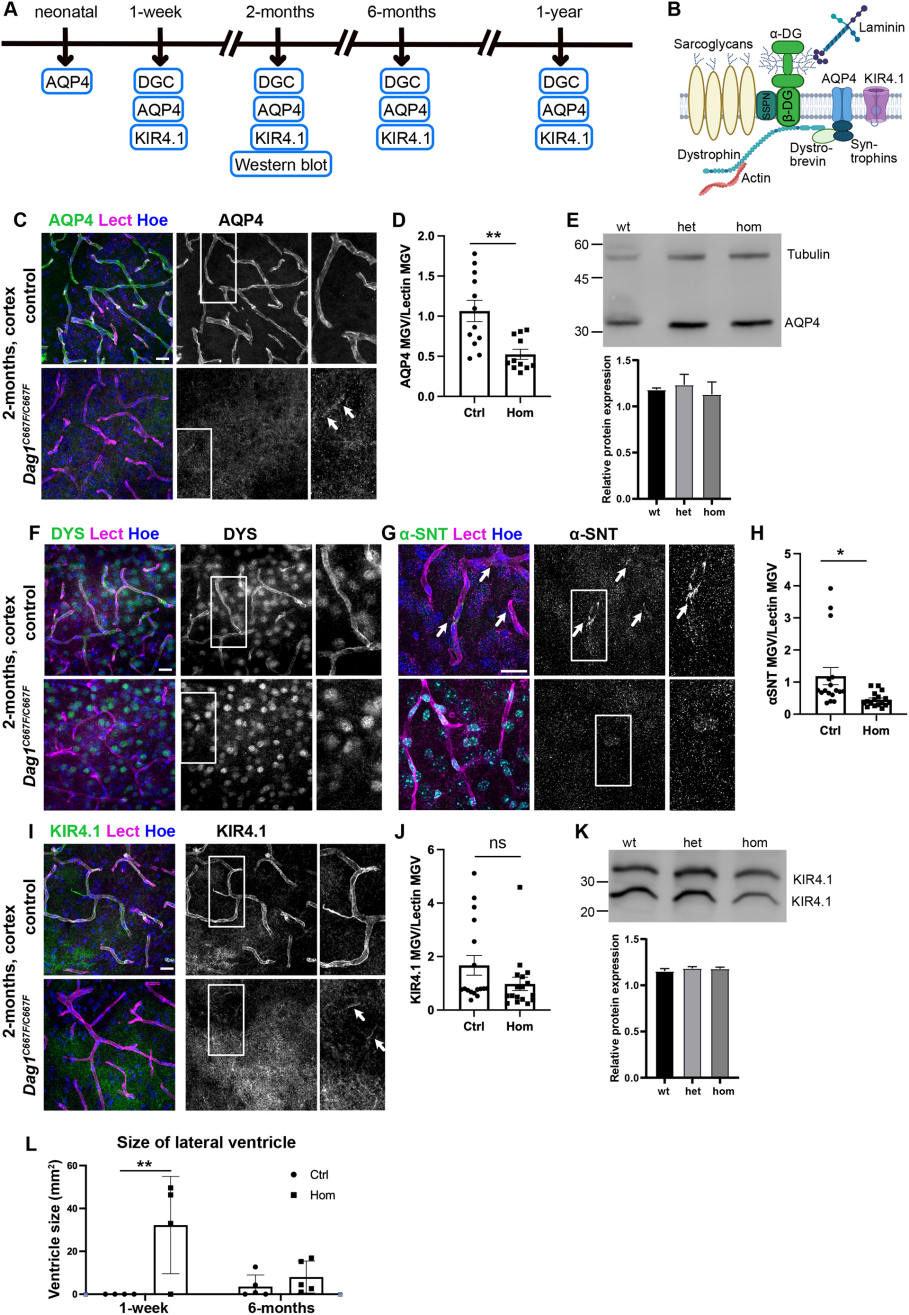
**The molecular composition of the glia-vascular unit is disrupted in the brain of *Dag1^C667F/C667F^* mice.** (A) Timeline summarizing which experiments were performed at which postnatal stages. (B) Schematic representation of the DGC at the BBB. SSPN, sarcospan; AQP4, aquaporin 4; KIR4.1, inwardly rectifying K^+^ channel. Created with Biorender.com. (C) Immunostaining for AQP4 in combination with lectin (Lect) and Hoechst (Hoe) in the cortex of 2-month-old mice. (D) Quantification of immunofluorescence intensity (mean gray value, MGV) of AQP4 in PVE of 2-month-old control (Ctrl) and *Dag1^C667F/C667F^* (Hom) mice. The MGV of AQP4 is normalized to the MGV of lectin. (E) Western blot for AQP4 using brain tissue of 2-month-old mice and quantification of relative protein levels. (F,G) Immunostaining for dystrophin (DYS) and α-syntrophin (α-SNT) in combination with lectin and Hoechst. (H) Quantification of immunofluorescence intensity of α-SNT in PVE of 2-month-old control and *Dag1^C667F/C667F^* mice. The MGV of α-SNT is normalized to the MGV of lectin. (I) Immunostaining for KIR4.1 in combination with lectin and Hoechst. KIR4.1 expression appears to be reduced in the PVE in the cortex of 2-month-old hom mutant mice. (J) Quantification of KIR4.1 immunofluorescence intensity in PVE of 2-month-old control and *Dag1^C667F/C667F^* mice. The MGV of KIR4.1 is normalized to the MGV of lectin. (K) Western blot for KIR4.1 using brain tissue of 2-month-old mice and quantification of relative protein levels. (C,F,G,I) Scale bars: 20 μm. (D,H,J) MGV analysis: *n*=6 Ctrl and 6 Hom animals; MGV was analyzed in three images per animal and included in the statistical analysis. Data are mean±s.e.m. Statistical analysis was performed using a two-tailed unpaired Student's *t*-test. (E,K) Quantification of protein bands is shown as an average (*n*=3 mice for each genotype) and normalized to tubulin levels (tubulin levels shown in E also correspond to the samples blotted in K). (L) Quantification of the ventricle size (average value of both ventricles) of 1-week- (*n*=4 per group) and 6-month-old (*n*=5 per group) mice. Data are mean±s.e.m. Statistical analysis was performed by two-way ANOVA with Sidak's multiple comparison test. *P<0.05, **P<0.01. ns, not significant.

To investigate whether the altered localization of DG results in aberrant localization of other proteins important for BBB function (Fig. 6), we examined the expression of aquaporin 4 (AQP4), which is highly enriched in the PVE and plays a crucial role in brain water homeostasis (Fig. 6A-E). AQP4 was no longer properly localized to the PVE in *Dag1^C667F/C667F^* mice and was detected only at very low levels around some blood vessels (Fig. 6C,D). Western blot analysis of brain tissue showed that the absence of AQP4 in PVE was not caused by an overall reduction in protein levels (Fig. 6E), suggesting that this phenotype is due to mislocalization of the water channel rather than to reduced expression levels. AQP4 is anchored to the PVE membrane by components of the DGC complex (Cherkaoui et al., 2021; Lien et al., 2012; Nicchia et al., 2008; Sato et al., 2018). Therefore, we next examined the localization of DYS and α-syntrophin (α-SNT) at the PVE. Immunostaining showed that both proteins were no longer localized to the PVE in the *Dag1^C667F/C667F^* mice at 2 months of age or older, suggesting that the C667F mutation results in altered interactions of β-DG with the intracellular components of the DGC (Fig. 6F-H). Finally, we examined the localization of the inwardly rectifying K^+^ channel KIR4.1 (also known as KCNJ10), another important component of the BBB that is also anchored to the PVE membrane by the DGC (Fujimoto et al., 2023; Jukkola and Gu, 2015). We found that the localization of KIR4.1 to the PVE appeared to be reduced in homozygous mutant mice when compared with controls, whereas the overall protein levels of KIR4.1 were not reduced in mutant animals (Fig. 6I-K). Interestingly, AQP4, α-SNT and KIR4.1 were still localized around blood vessels in the brain of 1-week-old homozygous animals (Fig. S4D-F), suggesting that DG is important for maintaining rather than establishing the molecular composition of the PVE during BBB maturation.

A consequence of a destabilized BBB can be the abnormal flow of cerebrospinal fluid and its accumulation in brain ventricles, resulting in ventricular enlargement (Profaci et al., 2020). Lateral ventricles of 1-week-old *Dag1^C667F/C667F^* mice were significantly enlarged compared with controls, suggesting a functional impairment of the BBB despite its apparently normal protein composition in the homozygous mice at this stage. However, no obvious difference in ventricle size could be detected between mutant and control mice at 6 months of age, pointing to possible compensatory mechanisms (Fig. 6L). In conclusion, these data indicate that the *Dag1^C667F^* mutation results in a specific impairment of DG function in the molecular organization of the PVE in the mouse brain with a potential impact on cerebrospinal fluid flow.

### The molecular composition of the glia-vascular unit is impaired in the retina of *Dag1^C667F/C667F^* mice

**Fig. 7.**
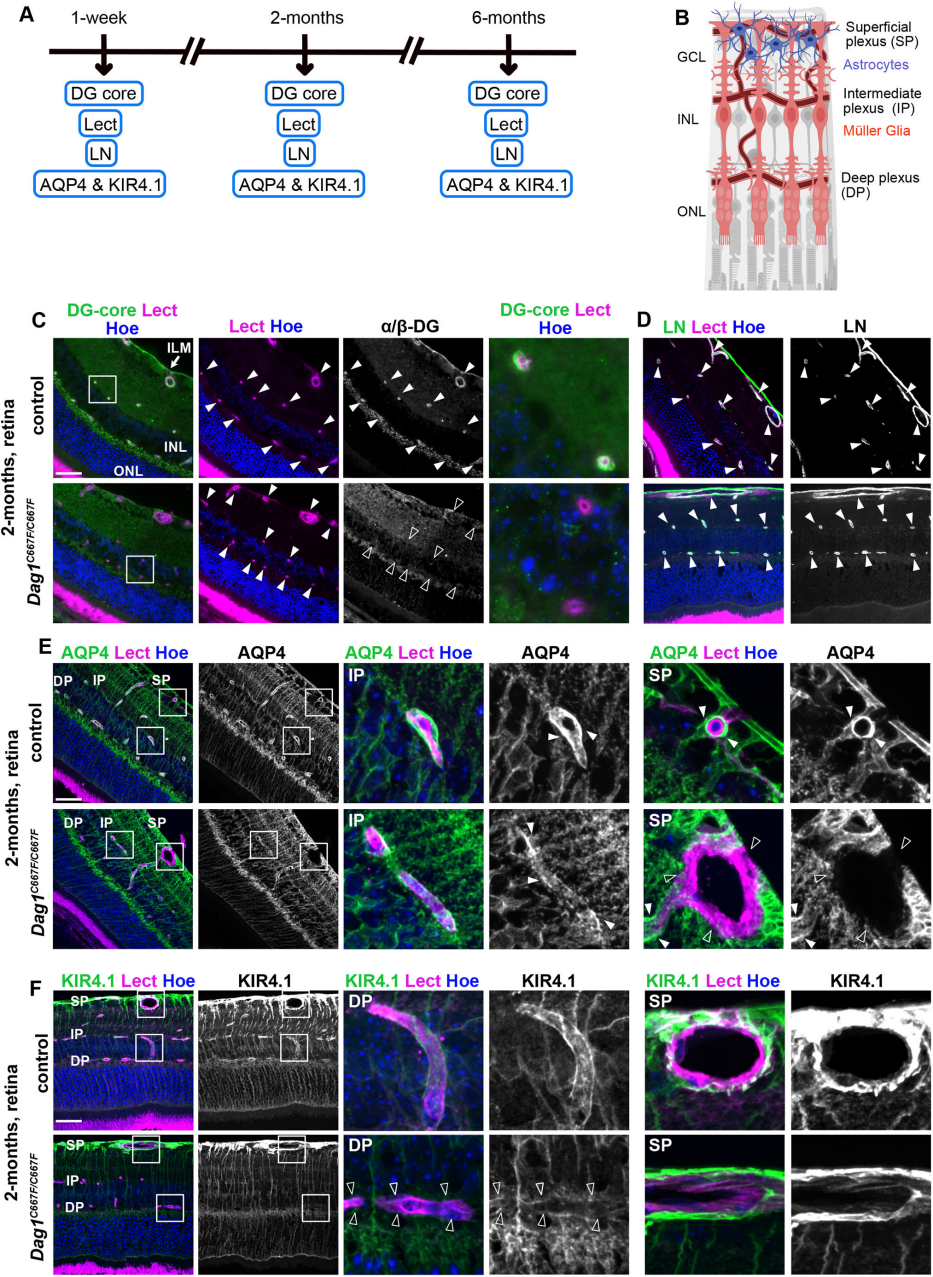
**The molecular composition of the glia-vascular unit is disrupted in the retina of *Dag1^C667F/C667F^* mice.** (A) Timeline summarizing which experiments were performed at which postnatal stages. (B) Schematic of the retina showing the vasculature, and the location of astrocytes and Müller glia cells. Created with Biorender.com. (C) Immunostaining for the DG-core protein (α/β-DG) in combination with lectin (Lect) and Hoechst (Hoe) in the retina of 2-month-old mice. Right panels show higher magnifications of the outlined areas in the left panels. Closed arrowheads indicate blood vessels (Lect Hoe) or α/β-DG surrounding these blood vessels (DG core). Open arrowheads indicate absence of DG around blood vessels. (D) Immunostaining for laminin (LN) in combination with Lect and Hoe in the retina of 2-month-old mice. (E) Immunostaining for AQP4 in combination with Lect and Hoe shows that AQP4 is absent around blood vessels in the superficial plexus (SP, open arrowheads) but maintained in the intermediate (IP) and deep plexus (DP) (closed arrowheads) in the retina of 2-month-old *Dag1^C667F/C667F^* mice. (F) Immunostaining for KIR4.1 in combination with lectin and Hoechst shows that KIR4.1 is reduced around blood vessels in the IP and DP (empty arrowheads) but maintained in the SP in the retina of 2-month-old *Dag1^C667F/C667F^* mice. (E,F) Areas outlined in the left panels are shown at higher magnification on the right. Scale bars: 40 μm.

Next, we examined the blood-retinal barrier at several postnatal stages (Fig. 7A). The blood-retinal barrier is formed by the PVE of Müller glia cells at blood vessels in the deep and intermediate plexus and by PVE of astrocytes at blood vessels in the superficial plexus (Fig. 7B) (Nicchia et al., 2016). The glial components of the blood-retinal barrier also contain DGC-AQP4-KIR4.1 complexes (Haenggi and Fritschy, 2006) and immunostaining for the DG core protein showed its localization around blood vessels in retinas of control animals. In the retinas of homozygous animals, the DG core protein was no longer detected around blood vessels (Fig. 7C). Immunostaining for glycosylated α-DG or β-DG could not be performed because the available antibodies did not work on paraffin wax-embedded sections of the retina. Laminin, which is part of the basement membrane around blood vessels in the retina (Gnanaguru et al., 2013), was not altered in homozygous mutant mice (Fig. 7D). Immunohistological analysis for AQP4 and KIR4.1 showed that in the retina of *Dag1^C667F/C667F^* mice, AQP4 was absent from the PVE at blood vessels in the superficial plexus, whereas KIR4.1 localization appeared to be normal or was only slightly reduced. In contrast, AQP4 expression persisted, albeit at reduced levels, in the PVE at blood vessels in the deep and intermediate plexus of homozygous mutant mice, whereas KIR4.1 was severely reduced (Fig. 7E,F). No difference in α-SNT localization, which appears to be diffuse in the retina and is not restricted to the blood-retinal barrier, was detected between control and mutant animals at 2 or 6 months of age (data not shown) (Enger et al., 2012). These data demonstrate that the mutation in β-DG has distinct effects on DGC-AQP4-KIR4.1 complex formation in the PVE formed by astrocytes versus Müller glia cells, consistent with previous data suggesting that the DGC-AQP4-KIR4.1 complex differs between these two glial cell types in the retina (Enger et al., 2012; Nicchia et al., 2016).

## DISCUSSION

### Partially penetrant embryonic lethality in *Dag1^C667F/C667F^* mice

*Dag1^C667F/C667F^* mice exhibit a severe developmental phenotype, with only about one-third of the homozygous mice surviving to term and most mutant embryos showing growth retardation by E9.5. A likely reason for this severe phenotype is that DG plays an essential role in maintaining Reichert's membrane, which is involved in placenta formation in rodents (Matsuo et al., 2022; Williamson et al., 1997). Indeed, the growth retardation in *Dag1^C667F/C667F^* embryos resembles the phenotype described for collagen IV-deficient embryos (Pöschl et al., 2004), where Reichert's membrane is thinner than in controls but not completely disrupted. Consistent with this, we find evidence for perturbed laminin expression in Reichert's membrane and decreased DG expression at the parietal endoderm/Reichert's membrane/trophectoderm interface in the mutant embryos. These data suggest that in embryos that fail to develop to term, DG expression levels or trafficking to the plasma membrane may be more severely affected than in homozygous mice that are born and subsequently live to adulthood. This could explain the relatively mild phenotypes (compared with those reported in human patients) observed in the group of surviving mutant mice. The mechanisms leading to this potential variability in DG levels or trafficking, and thus in embryonic survival, in *Dag1^C667F/C667F^* mice will be interesting to explore in the future, as they may be of therapeutic importance.

### DG expression levels and localization in the *Dag1^C667F/C667F^* mice and in human patients

Our results show reduced DG protein levels in adult *Dag1^C667F/C667F^* skeletal muscle and brain. However, α-DG glycosylation is unaffected and α/β-DG localization to the muscle fiber sarcolemma is not overtly altered in the homozygous mutant mice. This observation contrasts with reports on human patients in whom glycosylated α-DG was undetectable at the sarcolemma (Geis et al., 2013). Despite these differences between mouse and human, the reduction in DG levels in *Dag1^C667F/C667F^* mice suggests the following. First, a fraction of the unprocessed α/β-DG precursor may either be destroyed or form aggregates that are not transferable to gels or blotting membranes. Indeed, the clustering of β-DG observed at the PVE indicates aggregation. In this context, it is noteworthy that the C667F mutation leaves C711, which is thought to form an intramolecular disulfide bridge with C667, in the ectodomain of native β-DG (Deyst et al., 1995; Sciandra et al., 2012; Watanabe et al., 2007). Second, the presence of a reduced amount of properly processed, glycosylated and trafficked DG appears to be sufficient for adequate DG function in the development and maintenance of muscle, brain and eye. Third, although we cannot determine whether the observed phenotypes are solely due to reduced DG levels or whether altered structure and interaction capacity of β-DG contributes to the phenotype, it is important to note that DGC appears to remain intact in the skeletal muscle and functionally unimpaired in the radial glia scaffold during development, but is disrupted at the BBB and blood-retina barriers. These results suggest tissue-, cell- and perhaps even stage-specific consequences of the C667F mutation on DG function.

### Late-onset signs of muscle pathology and skeletal muscle dysfunction in *Dag1^C667F/C667F^* mice

Patients with the C669F mutation in *DAG1* have generalized muscular hypotonia and signs of muscular dystrophy (e.g. muscle fibers with moderate size variability) from early childhood (Geis et al., 2013). In the mouse model, some *Dag1^C667F/C667F^* males (6 months and older) show late onset signs of dystrophic muscle (increased percentage of fibers with central nuclei compared with control mice). This phenotype is not fully penetrant as it is not observed in all mutant male mice and is absent in female *Dag1^C667F/C667F^* mice. Despite this mild histopathological phenotype, 1-year-old male and female *Dag1^C667F/C667F^* mice were impaired in their performance on an activity wheel, particular in their running speed. These data indicate compromised muscle function, possibly because of reduced DG protein levels.

### Disruption of the DGC and associated proteins in PVE at the blood-brain and blood-retinal barrier of *Dag1^C667F/C667F^* mice

DGC, AQP4 and KIR4.1 localization in PVE is disrupted in *Dag1^C667F/C667F^* mice. AQP4 is known to be crucial for water homeostasis in the brain and retina. *Aqp4*-null mice have altered barrier function of PVE in the brain and the deep plexus of the retina, decreased blood-brain water uptake, and are more prone to develop a hydrocephalus (Haj-Yasein et al., 2011; Nicchia et al., 2016; Verkman et al., 2006). KIR4.1 colocalizes with AQP4 in the PVE. It has been proposed that the coupled transport of both water and K^+^ contributes to astrocytic volume changes after neuronal activity (Ochoa-de la Paz and Gulias-Cañizo, 2022).

It has previously been shown that the DGC is associated with AQP4 and plays an important role in the localization of AQP4 and KIR4.1 to PVE (Cherkaoui et al., 2021; Enger et al., 2012; Menezes et al., 2014; Nicchia et al., 2008; Rurak et al., 2007; Sato et al., 2018; Sene et al., 2009). For example, conditional inactivation of *Dag1* in the CNS results in greatly reduced localization of AQP4 at the PVE and an impaired BBB (Menezes et al., 2014). Loss of dystrophin protein 71 (Dp71), the major *Dmd* protein product in the adult brain and a direct interacting partner of DG, results in a strong reduction of AQP4 in PVE, a complete absence of α-SNT in the brain and a compromised blood-retinal barrier. In addition, β-DG localization in the PVE is reduced (Cherkaoui et al., 2021; Fujimoto et al., 2020; Sene et al., 2009). Dp71 interacts with both AQP4 and KIR4.1 in cortical astrocytes and in Müller glia cells, suggesting that the DGC is essential for the localization of both proteins in the PVE (Fujimoto et al., 2023; Sene et al., 2009).

The impaired BBB in *Dag1^C667F/C667F^* mice could lead to deficits in overall brain function. BBB disruption is emerging as an early biomarker of cognitive dysfunction in humans and has been proposed to contribute to age-related cognitive decline. In addition, prolonged BBB breakdown has been reported to be a key feature of COVID-associated brain fog. Mechanistically, a compromised BBB may trigger or exacerbate a range of tissue damage, leading to synaptic and neuronal dysfunction, and cognitive impairment (Greene et al., 2024; Hussain et al., 2021).

Taken together, the disruption of the molecular complexes at the PVE in brain and retina of *Dag1^C667F/C667F^* mice indicates that β-DG function is essentially lost in this highly specialized cell compartment of glia cells. This is in sharp contrast to the unimpaired development of the brain and retina, and to the mild muscle phenotype in the one-third of mutant mice that develop to term, suggesting that, despite the mutation, β-DG function is partially or fully maintained in these tissues or developmental stages. Importantly, the function of β-DG at the BBB may not be restricted to the PVE, as it is also expressed in endothelia cells and pericytes (our data and Zaccaria et al., 2001). Finally, the DGC has been shown to have several synaptic functions (Jahncke and Wright, 2023). Whether the C667F DG mutation also impairs β-DG function at CNS synaptic contacts and whether such a possible effect is direct or rather indirect, e.g. caused by the BBB disruption (see above), remains to be investigated.

### Other mouse models for dystroglycanopathies

Secondary dystroglycanopathies, which are characterized by severe neuromuscular pathologies, are caused by hypoglycosylation of α-DG due to genetic abnormalities in the enzymes responsible for the post-translational maturation of α-DG. The genes responsible for secondary dystroglycanopathies in human patients are *POMT1*, *FKTN*, *FKRP* and *POMGNT2*. When these genes are inactivated in mice, some mutations result in severe phenotypes, including embryonic lethality or perinatal death (Ackroyd et al., 2008; Kurahashi et al., 2005; Nakagawa et al., 2015; Willer et al., 2004), similar to the severe phenotype observed in the majority of *Dag1^C667F/C667F^* embryos. Interestingly, *myd* mice, which carry a mutation in the glycosyltransferase LARGE that is responsible for the synthesis of the laminin-binding tandemly repeated polymer of α-DG (i.e. matriglycan), are viable but have a shortened lifespan (Grewal et al., 2001).

Although numerous cases of secondary dystroglycanopathies have been described, few patients affected by primary dystroglycanopathies have been identified so far (Brancaccio, 2019). Besides the C669F mutation in *DAG1*, only three other mutations have been characterized in more detail. The observed phenotypes ranged from an early-onset form of limb-girdle muscular dystrophy (LGMD) with cognitive impairment (T192M mutation) (Dinçer et al., 2003; Hara et al., 2011) to a mild childhood-onset case of muscular dystrophy with hyperCKemia (V74I, D111N) (Dong et al., 2015) and a late-onset form of LGMD identified in a 64-year-old man (R776C) (Dai et al., 2019). The only available mouse model so far carries the missense mutation T190M (orthologous to the human T192M), which results in partially impaired glycosylation of α-DG (Bozic et al., 2004). The mouse model shows a late-onset muscular dystrophy phenotype (i.e. centrally located nuclei in skeletal muscle fibers in 21-week- and 1-year-old mice) (Hara et al., 2011). An effect on embryonic survival has not been reported. Thus, both the T190M and the C667F mouse models show a discrepancy in the onset and severity of the phenotype compared with the human patients.

### Implications of the *Dag1^C667F/C667F^* mouse model for the treatment of rare dystroglycanopathies

It should be noted that although the phenotype of the group of *Dag1^C667F/C667F^* mice that develop to term is less severe than the disease symptoms in human patients, the late onset histopathology in skeletal muscle, along with abnormalities in the PVE in the brain and retina, may well be characteristic of the mild end of the MEB disease spectrum, making this mouse model a valuable tool for studying certain mechanistic aspects of this primary dystroglycanopathy. In particular, the patients with the *DAG1* C669F mutation present with megalencephalic leukoencephalopathy with subcortical cysts (MLC) (Geis et al., 2013). In the majority of patients with MLC, the phenotype is associated with pathogenic variants in *MLC1* or *GLIALCAM* (*HEPACAM*), but recently, in MLC patients without mutations in these two genes, a pathogenic variant of *AQP4* was discovered that interferes with the membrane localization of AQP4 (Passchier et al., 2023). Thus, our finding that AQP4 localization to PVE is disrupted in *Dag1^C667F/C667F^* mice may provide a first insight into the potential mechanisms underlying the MLC in the patients with the C669F mutation in *DAG1*. A potentially directly relevant therapeutic approach in the context of the PVE phenotype in our mouse model is the observation that AQP4 polarization in PVE is altered in a rat retinal injury model. This deficit can be rescued by treatment with bumetanide, a NKCC1 (Na^+^-K^+^-2Cl^–^ co-transporter 1) inhibitor that downregulates AQP4 by interfering with the metalloproteinase 9 (MMP9)-mediated cleavage of β-DG (Chen et al., 2023). In addition, a better knowledge of the DG core protein can guide the design of better antibodies, especially for diagnostic purposes (Fortunato et al., 2014; Humphrey et al., 2014). Thus, we anticipate that the availability of the *Dag1^C667F/C667F^* mouse model for further molecular or pharmacological studies may have a biomedical impact.

## MATERIALS AND METHODS

### Animals

All mouse experiments were carried out with strict observance of protocols and guidelines approved by the University of Bonn Animal Care and Use Committee, Federal Government of Germany and European Union legislation. The protocols were approved by the Landesamt für Natur, Umwelt und Verbraucherschutz Nordrhein-Westfalen (Permit numbers: 81-02.04.2019.A415, 81-02.04.2019.A493). The mice were housed under controlled light (12:12 h light:dark cycle at an ambient temperature of 22°C). Water and mice chow were available *ad libitum*. For embryonic stages, 12:00 noon on the day of the vaginal plug was designated as E0.5.

### Generation of mouse line

The *Dag1^C667F^* mouse line was developed by Genoway (Lyon, France) using a standard homologous recombination approach in embryonic stem cells. To generate the C667F mutation (corresponding to the C669F mutation in human) a point mutation (TGC>TTC at position 2698 in the cDNA sequence) was introduced into exon 5. The targeting vector containing the mutated exon 5 and flanking genomic regions (homology arms) was transfected into mouse ES cells. Neomycin (Neo)-resistant cells were screened by PCR and DNA sequencing. Positive clones were injected into blastocysts to generate chimeras. To excise the Neo cassette and generate the F1 generation (heterozygous point mutant knock-in mice), chimeras were crossed with mice expressing Cre in the germline. Pups from the F1 generation were then screened to test for germline transmission. Mice were generated on a C57BL6 background and then crossed onto a CD1 background. Heterozygous mice were crossed to obtain homozygous *Dag1^C667F/C667F^* mice.

### Genotyping

PCR amplification was performed to identify wild-type, heterozygous and homozygous mice. Genomic DNA was isolated from extra-embryonic membranes (E9.5 and E10.5), embryos (E8.5), tail tips (neonates) or ear clips using sodium hydroxide digestion (digestion with 75 μl NaOH for 1 h at 96°C followed by neutralization with pH 5.5 Tris HCl). PCR amplification was carried out using these conditions: 95°C for 30 s, 65°C for 30 s and 72°C for 30 s (30 cycles), with a final extension at 72°C for 8 min. The PCR products were loaded onto 1.5% agarose gel with ethidium bromide and photographed using a ChemiDoc and Image Lab software. Primers were: forward, 5′-CCCCAGACTGGCCTTCAACTCATC-3′; reverse, 5′-AGTGCCCTATCACATGACATCCTGTCAC-3′. The wild-type *Dag1* allele yields a 177 bp product, the C667F *Dag1* mutant allele a 268 bp product.

### Histology

#### Tissue preparation at embryonic stages

Pregnant mice were euthanized by cervical dislocation at the appropriate embryonic stage. E8.5 embryos were kept in their extra-embryonic tissues, fixed overnight in 4% paraformaldehyde (PFA) at 4°C, dehydrated in graded series of ethanol followed by xylene, embedded in paraffin wax and sectioned at 7 μm on a microtome. E9.5 and E10.5 embryos were dissected from extra-embryonic tissues, the embryos and extra-embryonic tissues were fixed separately in 4% paraformaldehyde (PFA) overnight, dehydrated in graded series of ethanol followed by xylene, embedded in paraffin wax and the tissues were sectioned at 7 μm on a microtome.

#### Tissue preparation of postnatal brain, muscle and eye

Postnatal day (P) 0 (neonatal) and P7 (1 week) mice were decapitated; P60 (2 months), P180 (6 months) and 1-year-old mice (age range: 1.05 to 1.25 years) were euthanized using cervical dislocation. Eyes, brains and whole hindlegs (for neonates) or hindleg muscles (for mice 1 week and older: upper and lower extensors, upper and lower flexors) were dissected. Eyes were fixed overnight in 4% PFA, dehydrated in graded series of ethanol followed by xylene, embedded in paraffin wax and cut into 5 μm sections on a microtome. Brains were fixed in 4% PFA, cryoprotected in 15% and 30% sucrose, and cryopreserved in OCT Tissue Tek (Sakura) on dry ice. P0 and P7 brains were cut into 14 μm sections onto adhesive microscope slides, while brains from P60 and older mice were cryosectioned at 40 µm and collected as free-floating sections in anti-freeze solution (40% PBS, 30% ethylene glycol and 30% glycerol). Mouse muscle or hindlegs were snap frozen in isopentane chilled in liquid nitrogen and cryosectioned at 7 μm. For 1-year-old mice, muscles, brain and eyes were isolated from animals that had access to a running wheel for 8 days before the tissues were collected.

#### Hematoxylin and Eosin staining

Paraffin sections of eye tissue were deparaffinized and rehydrated. Frozen sections were thawed and hydrated in PBS (brain tissue) or stained directly (muscle tissue). Sections were stained for 3 min in Hematoxylin, differentiated for 5 s (muscle) or 30 s (brain and eye) in 0.2% HCl and 75% ethanol, rinsed for 5 min in running tap water, washed in double-distilled water for 1 min and stained with Eosin for 3-5 min. Sections were then dehydrated in increasing concentrations of ethanol followed by xylene and mounted with DPX (Merck) non-aqueous mounting medium.

### Immunostaining

For immunofluorescence staining of frozen sections, sections were re-fixed in 4% PFA for 10 min at room temperature and blocked in 10% NDS in PBS plus Triton X-100 (PBT) for 1 h at room temperature. For blocking and all the following steps, 0.1% PBT was used for P0 and P7 brain tissue and muscle tissue at all stages, whereas 0.3% PBT was used for adult brain tissue. Sections were incubated with primary antibody in 3% NDS PBT overnight at 4°C. To visualize brain endothelial cells, DyLight649 *Lycopersicon esculentum* lectin was added to the primary antibody solution (1:200; Vector Labs). Sections were washed three times for 5-10 min in PBT and incubated for 2 h at room temperature in secondary antibody and Hoechst (Abcam) in 3% NDS in PBT. Sections were washed three times for 5-10 min in PBT. Antibody details are provided in Table S1.

For immunofluorescence staining using the anti-β-DG antibody (mouse, Novocastra), a ‘mouse on mouse’ protocol was used. Sections were incubated in 10% NGS in 0.1% PBT for 30 min at room temperature. Sections were then incubated with unconjugated Fab fragments (Rockland) diluted at 1:100 in 1% BSA in 0.1% PBT for 1 h. Sections were washed three times for 5-10 min in 0.1% PBT followed by the standard immunofluorescence protocol described above but using DyLight549-conjugated goat-anti-mouse F(ab′)_2_ fragments as secondary antibody. Antibody details are provided in Table S1.

For immunofluorescence staining of paraffin sections, sections were deparaffinized and rehydrated. Antigen retrieval was carried out by boiling the sections for 10 min in sodium citrate buffer (pH 6.0) in a pressure cooker. Sections were blocked for 1 h at room temperature with 10% NDS in 0.3% PBT and incubated overnight at room temperature with primary antibodies in 3% NDS in 0.3% PBT. To visualize brain endothelial cells, DyLight649 *Lycopersicon esculentum* lectin was added to the primary antibody solution (1:200; Vector Labs). Sections were washed three times for 5-10 min in 0.3% PBT and incubated with secondary antibodies and Hoechst (Abcam) in 3% NDS in 0.3% PBT for 1 h at room temperature. Sections were washed three times for 5-10 min in 0.3% PBT and mounted with Aqua Polymount (Polysciences). Antibody details are provided in Table S1.

### Protein isolation and western blot

Proteins were extracted from skeletal muscle (hindleg hip adductor and abductor, and thigh knee flexor complexes) and from the whole brain of wild-type, heterozygous and homozygous mice (2 months, 6 months and 1 year) using PBS containing 1% Triton X-100 (Sigma-Aldrich) and a Complete EDTA-free cocktail of protease inhibitors (Roche). Soluble proteins from skeletal muscle were incubated with Protein-A beads. The cleared fractions of skeletal muscle samples and total protein extracts of the brain were incubated with 500 μl of succinylated WGA-agarose beads (Vector Labs) at 4°C for 16 h. Beads were washed three times in washing buffer (WB, 1 ml PBS containing 0.1% Triton X-100 and protease inhibitors) and eluted with 300 μl WB containing 300 mM N-acetylglucosamine. Total protein extract or WGA-enriched proteins (20 μg) were separated using 4-15% SDS-PAGE gels and were transferred to nitrocellulose membrane (Millipore). Blots were probed with primary antibodies and then developed with horseradish peroxidase (HRP)-enhanced chemiluminescence (WesternBright ECL). Antibody details are provided in Table S1.

### RNA isolation and quantitative RT-PCR

Total RNA was isolated from whole brain of 1-year-old wild-type, *Dag1^C667F/+^* or *Dag1^C667F/C667F^* mice using the RNeasy Mini Kit (Qiagen) according to the manufacturer's instructions. An additional on-column DNase treatment was performed to remove residual DNA. 1 μg of total RNA was reverse transcribed in a 20 μl reaction mix using the High-Capacity cDNA Reverse Transcription Kit (Applied Biosystems), following the manufacturer's instructions. Quantitative RT-PCR was performed using a standard TaqMan PCR protocol on a StepOne real time PCR System (Applied Biosystems) with primers specific for murine *Dag1*. The housekeeping gene hypoxanthine phosphoribosyltransferase (*Hprt*) was used as a reference. The reactions were incubated at 50°C for 2 min and at 95°C for 10 min, followed by 40 cycles of 95°C for 15 s and 60°C for 1 min. All reactions were run in triplicate.

### Imaging

Whole-mount images of E9.5 and E10.5 embryos were captured with a Leica MZ10 F modular stereomicroscope and processed using Leica Application Suite Version 3.3.0 (Leica Microsystems, 2003). Images of immunofluorescence-stained sections were acquired on an inverted Zeiss AxioObserver Z1 equipped with a Zeiss AxioCam MRm. At 5× (EC PlnN 5×/0.16), 10× (EC PlnN 10×/0.3) and 20× (EC PlnN 20×/0.5) magnification, tile images were acquired with conventional epifluorescence. Tile images were stitched with Zen blue software (Zeiss, 2012).

At 63× (C-Apochromat, 63×/1.4 oil, Zeiss), images of immunofluorescence stainings were obtained at an inverted Zeiss AxioObserver equipped with a CSU-W1 Confocal scanner unit (50 mm pinhole disk, Yokogawa). *Z*-stacks were acquired with laser lines 405 nm, 488 nm, 561 nm and 640 nm. Images taken are maximum intensity projections of these *z*-stacks.

Super-resolution 3D structured illumination microscopy was performed on an OMXv4 (GE Healthcare) equipped with a 60×/N.A. 1.42 Olympus oil immersion lens and four separate 15-bit sCMOS cameras for fluorescent channel imaging. The corresponding laser excitation wavelengths were 405, 488, 568 and 642 nm. The raw images were processed with SoftWoRx 7.0.0 to construct 3D super resolution images. The 3D reconstruction was obtained with the molecular visualization software ChimeraX (Pettersen et al., 2021).

Bright-field images were acquired on a Zeiss Axio Scope.A1 microscope using an AxioCam 503 Color and processed with Zen blue software (Zen lite, 2019).

### Voluntary wheel running

One-year-old mice (age range: 1.05 to 1.25 years; males *n*=4 and females *n*=2 per group) were individually housed in cages with an activity wheel (Scurry Mouse Misstep Wheel, Lafayette Instrument) for a period of 8 days. Mice were provided with free access to food and water, as well as nesting material, and were allowed to run on the wheel at any intensity or duration. Mice initially had access to a 33-rung wheel (regular wheel) for 2 days. The complexity of the activity wheel was then increased according to the following scheme: mice were housed in a cage with an irregularly spaced 22-rung wheel (complex wheel) for 4 days and with an irregularly spaced 14-rung wheel (highly complex wheel) for 2 days. The running activity was recorded using Scurry Activity Monitoring Software (Lafayette Instrument) and through video recordings throughout the experimental period. Nocturnal (dark period in the light cycle) running activity was tallied and maximum speed, average speed, total duration per night and total distance per night were calculated using Microsoft Excel (version 16.16.27, Microsoft). Nocturnal running sessions, defined as a period of uninterrupted running on the wheel, were analyzed using Igor Pro software (version 8.04, Wavemetrics) for average speed, maximum speed, average duration and average distance per session, as well as for the total number of sessions per night.

### Quantification

#### Tissue sections

Cross-section areas and minimal Feret's diameters in quadriceps femoris muscle fibers of neonatal, 1-week-, 6-month- and 1-year-old (age range: 1.05 to 1.25 years) mice were measured to quantify muscle fiber size variability as a surrogate for muscle fiber degeneration and regeneration. Bright-field images of Hematoxylin and Eosin-stained sections were acquired with a 10× (6-months, 1-year) or a 20× (neonatal, 1-week) objective, muscle fibers were traced and the cross-sectional area and minimal Feret's diameter were quantified using ImageJ (Treat-NMD SOP DMD_M.1.2.001 Version 2.0). The percentage of muscle fibers with centralized myonuclei was also calculated for neonatal, 1-week-, 6-month- and 1-year-old mice to objectify muscle fiber regeneration. Muscle fibers with centralized myonuclei were manually counted using 10× Hematoxylin and Eosin light microscope images, and expressed as a percentage of all muscle fibers in the image.

Levels of α- or β-DG expression at the sarcolemma of muscle fibers were determined in quadriceps femoris muscle fibers from 1-year-old mice (*n*=4 per group). In maximum intensity projections of *z*-stacks (63× objective) acquired from α- or β-DG immunofluorescence-stained sections, binary masks were used to generate ROIs of the sarcolemma, and the mean gray values (MGVs) in the ROIs were measured using ImageJ.

To quantify β-DG expression in the SR of muscle fibers, immunofluorescence staining for the SR marker CASQ and β-DG was performed on the quadriceps femoris muscle of 6-month-old mice (*n*=5 per group). In maximum intensity projections of *z*-stacks (63× objective) acquired from immunofluorescence-stained sections, binary masks based on CASQ were used to generate ROIs of the SR, and the MGV of β-DG staining was measured in the ROIs using ImageJ.

Brain ventricle size in 1-week-old (*n*=4 per group) and 6-month-old (*n*=5 per group) mice was measured by immunofluorescence microscopy. Images of the mouse brain cortex at the level where the lateral ventricles are widest were acquired with a 5× objective. The ventricle size was manually traced, and the area of each ventricle was measured in ImageJ. The average of the left and right ventricle size was calculated.

To quantify protein expression at the PVE, mean gray values of immunofluorescence-stained blood vessels (α- and β-DG, KIR4.1, AQP4 and α-SNT) were measured in cerebral cortex and retina of 2-month-old (*n*=5 per group) and 6-month-old (*n*=5 per group) mice. Using 63× maximum intensity projections of *z*-stack images, blood vessels were manually traced based on Lectin staining, and the mean gray values of the traced areas were measured using ImageJ. Mean gray values for the marker of interest were normalized to the mean gray value of lectin.

#### Western blots

The densitometry analysis of bands was performed using Alliance Q9 Advanced UVITEC software and normalized to tubulin.

#### Quantitative RT-PCR

The relative level for each gene was calculated using the 2^−ΔΔCT^ method (Livak and Schmittgen, 2001) and reported as fold change. Each experiment was repeated three times.

### Methodology and statistics

Sample size was not determined in advance, as we had to work with a relatively small sample size due to embryonic lethality in two-thirds of the homozygous animals. All homozygous animals were included in the analysis. For the analysis of postnatal stages, a similar number of mutant and control animals was analyzed; thus, not all control animals were included into the analysis. There were no pre-established criteria to include or exclude control animals. The sample phenotypes were not anonymized.

#### Western blots

One-way ANOVA followed by Sidak's multiple comparison test was used for normalized densitometric measurement of control and mutant mice using GraphPad Prism 8.0 software. Error bars indicate s.d.

#### Quantitative RT-PCR

One-way ANOVA followed by Sidak's multiple comparison test was used on GraphPad Prism 8.0 software.

#### Histology and immunostaining

Two-tailed unpaired Student's *t*-test was used for the statistical analysis of mean gray values of blood vessels. One-way ANOVA with Sidak's multiple comparison test was used for analysis of mouse weights on GraphPad Prism software 9.5. Histograms for muscle fiber cross-section area and minimal Feret's diameter were plotted. VCs of muscle fiber cross-section area and minimal Feret's diameter were calculated using the formula VC=1000×standard deviation/mean (Briguet et al., 2004). VCs were compared using one-way ANOVA with Sidak's multiple comparison test. Statistical tests were performed in GraphPad Prism software 9.5 and 10.0.3.

#### Voluntary wheel running

Two-way ANOVA with Sidak's multiple comparison test was used for analysis of maximum speed per night and per session, average speed per night and per session, total duration per night, total distance per night, average session duration and average session distance. Statistical tests were performed in GraphPad Prism software 9.5 and 10.0.3. Error bars indicate s.e.m. or s.d., as specified in the figure legends.

## Supplementary Material

10.1242/dmm.050594_sup1 Supplementary information
