# Supplementary material for: The missense mutation C667F in murine β-dystroglycan causes embryonic lethality, myopathy and blood-brain barrier destabilization
Source: Dis Model Mech. 2024 Jun 18;17(6):dmm050594. doi: 10.1242/dmm.050594 (PMC11212641; doi:10.1242/dmm.050594)
Supplement: Supplementary information [file dmm-17-050594-s1.pdf]

Figure S1

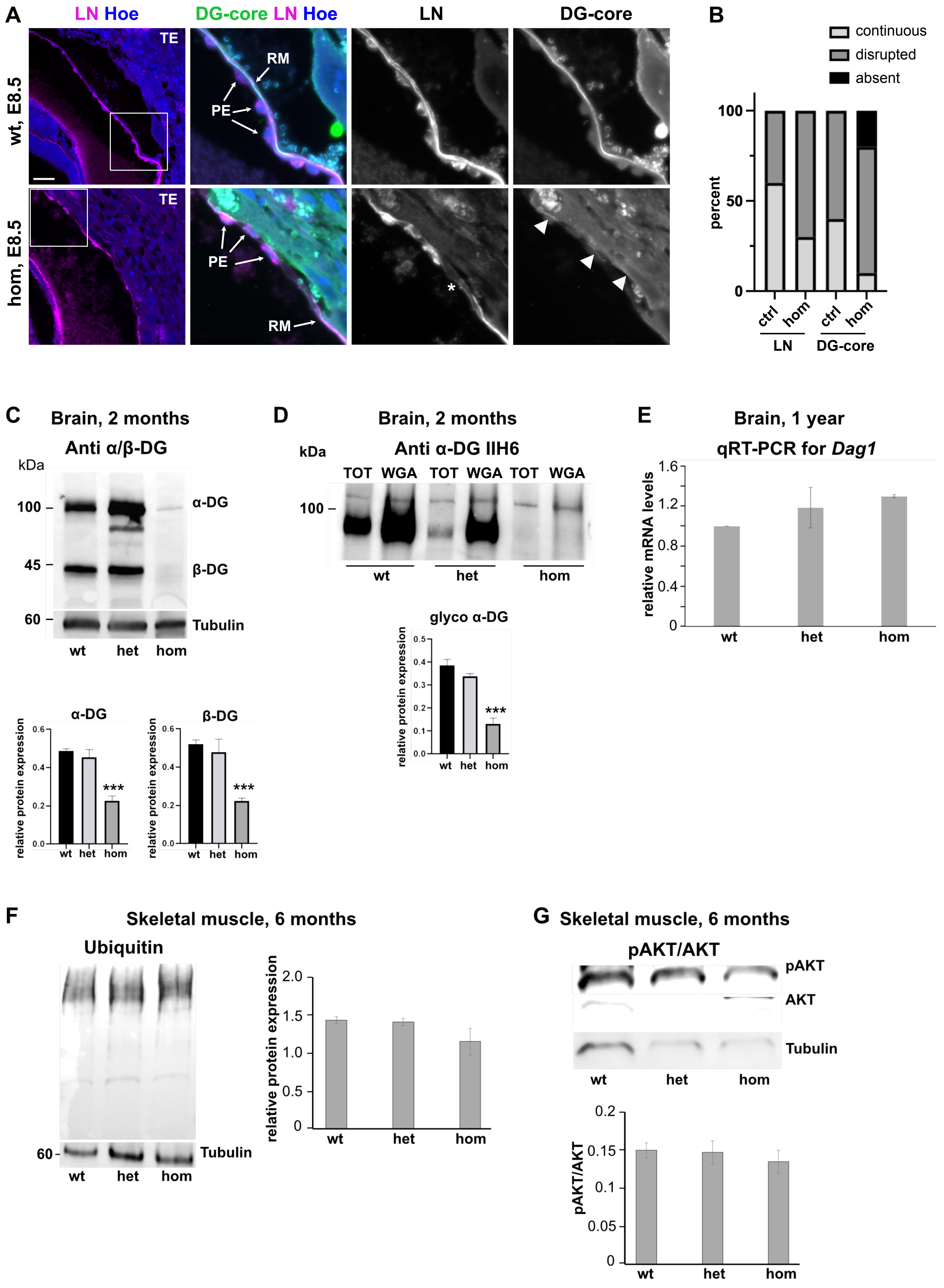

Disease Models & Mechanisms • Supplementary information

Fig. S1. Analysis of Reichert's membrane in embryos and DG protein levels in brain tissue of *Dag1*$^{C667F/C667F}$ mice

(A, B) Analysis of Reichert's membrane in E8.5 control (wild-type (wt) and heterozygous (het)) or homozygous (hom) embryos. (A) Immunostaining for Laminin (LN) and the DG-core protein in combination with Hoechst (Hoe) to visualize cell nuclei. In control (ctrl) embryos, LN is deposited evenly in Reichert's membrane (RM) and DG is expressed at the parietal endoderm (PE)/RM/trophectoderm (TE) interface. Note that the RM is detached from the TE in the wt embryo, which is likely due to processing artifacts. In hom embryos, LN is unevenly distributed in RM (asterisks) and DG is only weakly expressed at the PE/RM/TE interface (arrowheads). Scale bar: 50 μm. (B) Qualitative assessment of continuity of LN expression in RM and DG expression at the PE/RM/TE interface in E8.5 embryos. Ctrl embryos: n=6, hom embryos: n=7. (C,D) DG expression was assessed by Western blot performed on brain samples from wt, het and hom mice at 2 months of age (n=3 for each genotype). Densitometric analysis of protein bands is shown as DG/Tubulin ratio. (C) α- and β-subunits were analyzed in total protein extracts using a polyclonal anti α/β-DG antibody that recognizes both core proteins. (D) α-DG was detected in WGA enrichments with the IIH6 monoclonal antibody, which is specific for a glycosylated epitope of α-DG. Error bars: s.d.. Statistical analysis was performed by one-way ANOVA followed by Sidak's multiple comparison. ***$p<0.001$ (hom compared to wt). (E) Quantitative RT-PCR was performed on cDNA amplified from brain tissue of 1- year-old mice. Quantification of mRNA level is normalized to wt mRNA level and shown as average (n=3 mice per genotype). Error bars: s.d.. (F, G) Analysis of skeletal muscle tissue (hind limb hip adductor and abductor, and thigh knee flexor complexes) from 6-month-old mice (n=3 mice per genotype). (F) Densitometric analysis of protein ubiquitination in WGA enriched samples and representative Western blot image of ubiquitination. (G) Densitometric analysis of pAKT and AKT expression shown as ratio between pAKT and AKT in total protein extracts and representative Western blot images of phosphorylated and total Akt.

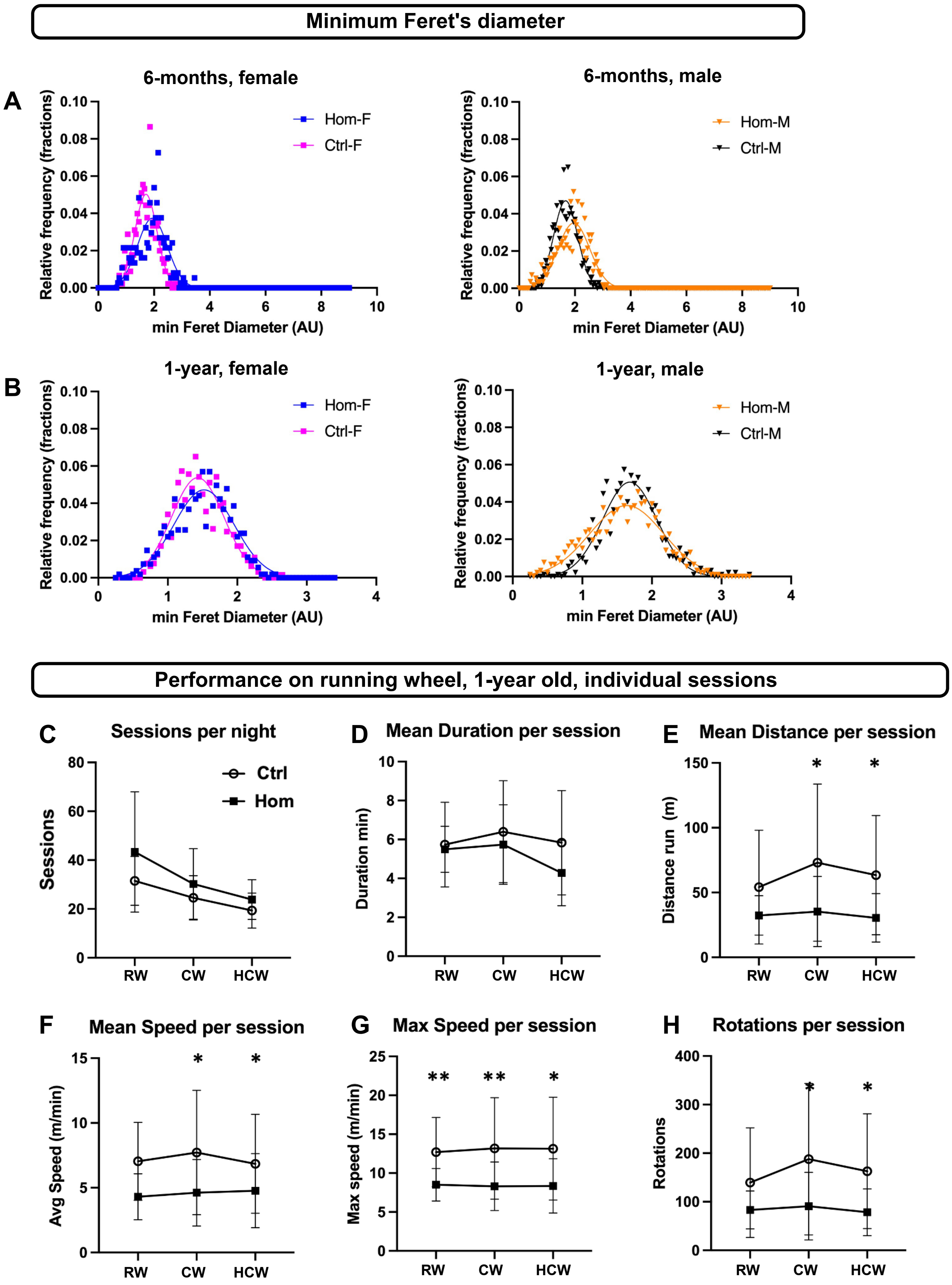

Minimum Feret's diameter
A
6-months, female
Hom-F
Ctrl-F
Relative frequency (fractions)
min Feret Diameter (AU)
6-months, male
Hom-M
Ctrl-M
B
1-year, female
1-year, male
Performance on running wheel, 1-year old, individual sessions
C
Sessions per night
Ctrl
Hom
Sessions
D
Mean Duration per session
Duration min)
E
Mean Distance per session
Distance run (m)
F
Mean Speed per session
Avg Speed (m/min)
G
Max Speed per session
Max speed (m/min)
H
Rotations per session
Rotations
RW
CW
HCW

Fig. S2. Histopathological phenotype of muscle fibers and impaired wheel running in *Dag1*$^{C667F/C667F}$ mice

(A, B) Histograms of minimum Feret's diameter of mice at 6 months (n=5 per group) and 1 year (n=6 per group) of age show a broadening and flattening of the distribution of variance coefficients (VCs) in female and male homozygous mice (Hom-F, Hom-M) compared to controls (Ctrl-F, Ctrl-M). (C-H) Running wheel performance per session. There is no difference in the number or duration of sessions on the running wheel between *Dag1*$^{C667F/C667F}$ mice (Hom) and controls (Ctrl) (C, D). Maximum speed per session is significantly reduced in Hom mice compared to Ctrls on the regular wheel (RW), complex wheel (CW), and highly complex wheel (HCW) (G, see also Figure 3E). Correspondingly, mean distance per session (E), mean speed per session (F) and rotations per session (H) are also significantly reduced when comparing the Hom mice to Ctrls (only for CW and HCW). n=6 mice per group. Error bars: s.e.m.. Statistical analysis was performed by two-way ANOVA with Sidak's multiple comparison. *$p<0.05$; **$p<0.01$.

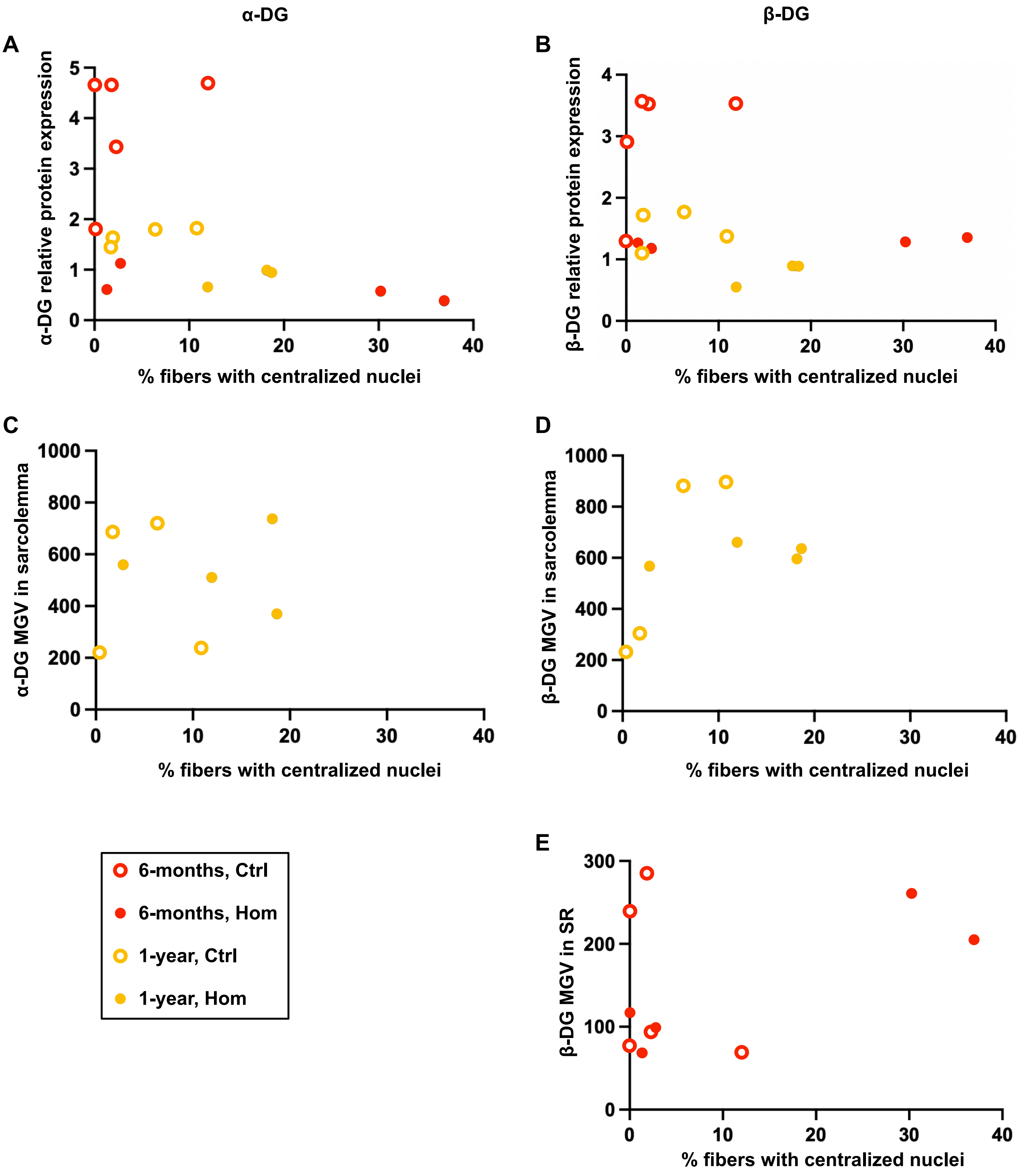

Disease Models & Mechanisms • Supplementary information

Fig. S3. No clear correlation between histopathological phenotype and α-DG or β-DG protein expression level or subcellular localization

(A, B) There is no obvious correlation between the expression levels of α-DG or β-DG as detected by Western blot analysis and the percentage of central nuclei in individual 6-month- or 1-year-old homozygous (Hom) or control (Ctrl) mice. (C, D) There is no obvious correlation between the intensity (measured by mean gray value (MGV)) of the immunofluorescence signal for α-DG or β-DG in the sarcolemma and the percentage of central nuclei in individual 1-year-old Hom or Ctrl mice. (E) There appears to be a potential correlation between the intensity (measured by MGV) of the immunofluorescence signal for β-DG in the sarcoplasmic reticulum (SR) and the percentage of central nuclei 1-year-old Hom mice, but not in Ctrl mice.

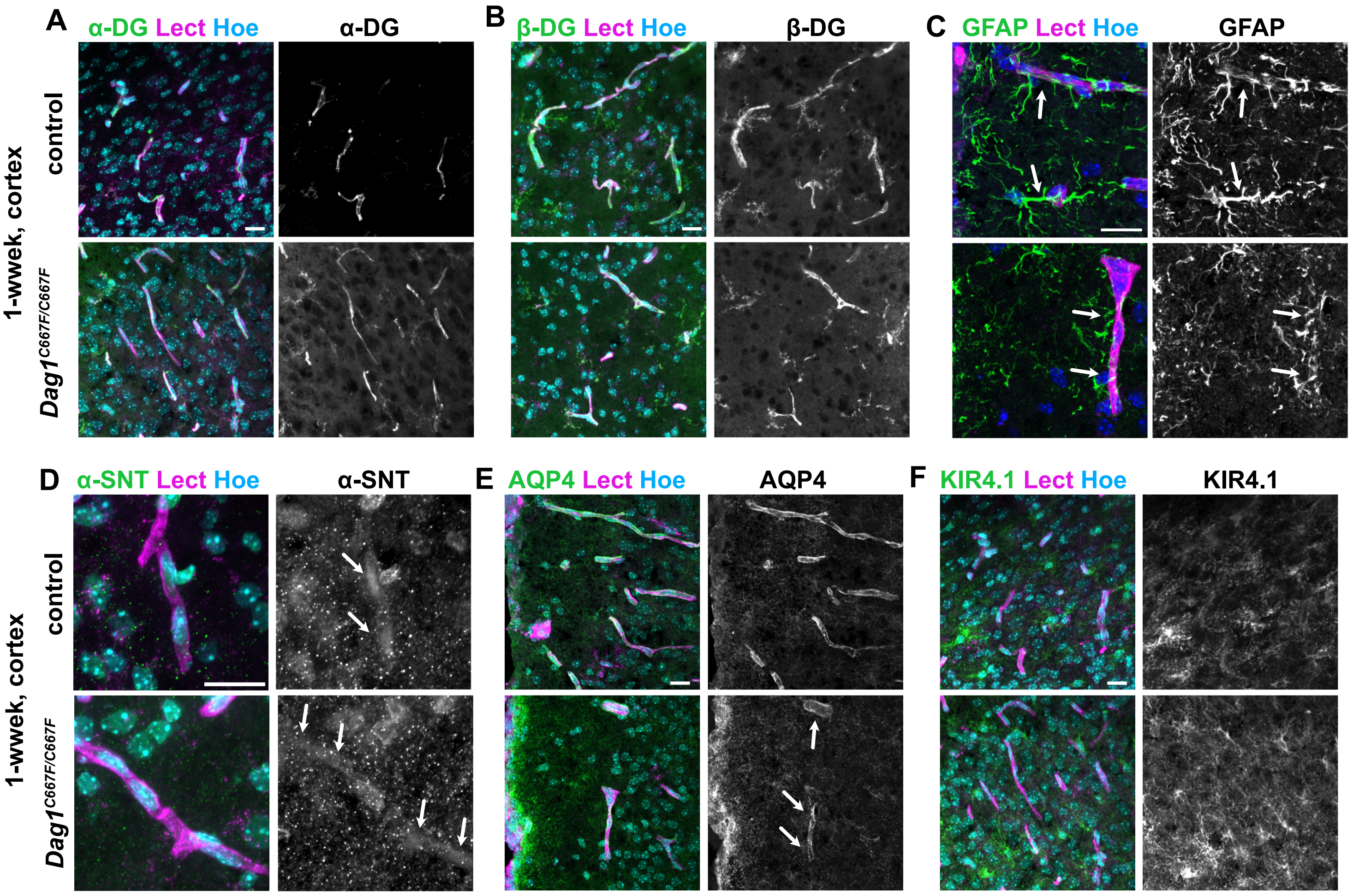

Fig. S4. The molecular composition of the glia-vascular unit is normal in the brain of 1-week-old *Dag1*$^{C667F/C667F}$ mice

Immunostaining for glycosylated (glyco) α-DG (A), β-DG (B), GFAP (C), α-syntrophin (α-SNT, D), Aquaporin 4 (AQP4, E) and KIR4.1 (F) in combination with lectin (Lect) to visualize blood vessels and Hoechst (Hoe) to visualize cell nuclei in the cortex of 1-week-old mice. Note that in contrast to 2-month-old mice (compare Figure 6) all the markers are present in the perivascular endfeet at this early postnatal stage. Scale bars: 20 µm.

Disease Models & Mechanisms • Supplementary information

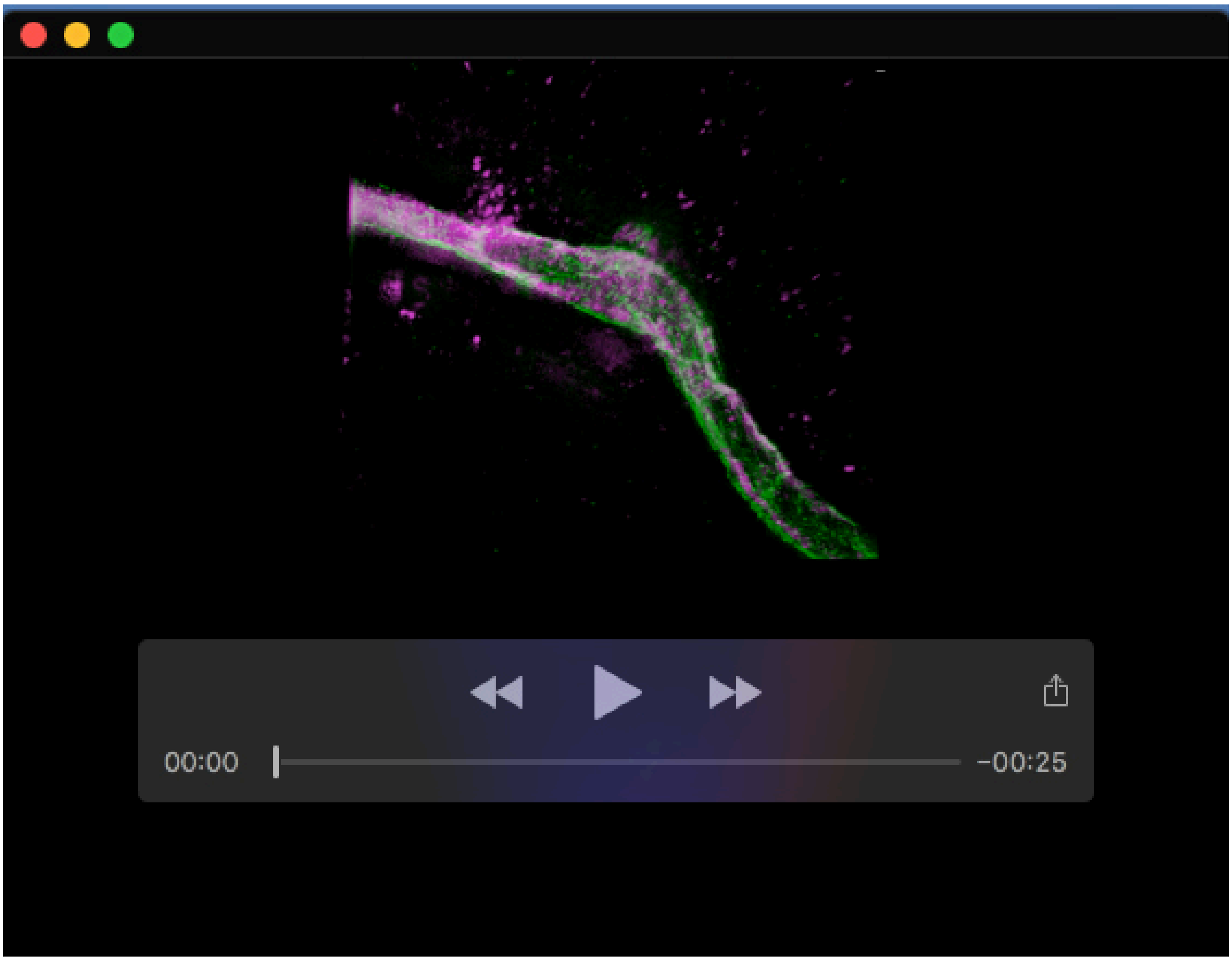

Movie 1. Super-resolution 3D structured illumination microscopy image showing the localization of β-DG in the perivascular space in the cortex of control mice. Immunostaining for β-DG (green) in combination with lectin (magenta) to visualize blood vessels and Hoechst (cyan) in the cortex of 2-month-old control mice. 3D reconstruction of a 40.96 x 40.96 x 3 μm z-stack. Note that there are no nuclei in the selected region in the control. This region of interest is also shown in Figure 5E.

Disease Models & Mechanisms • Supplementary information

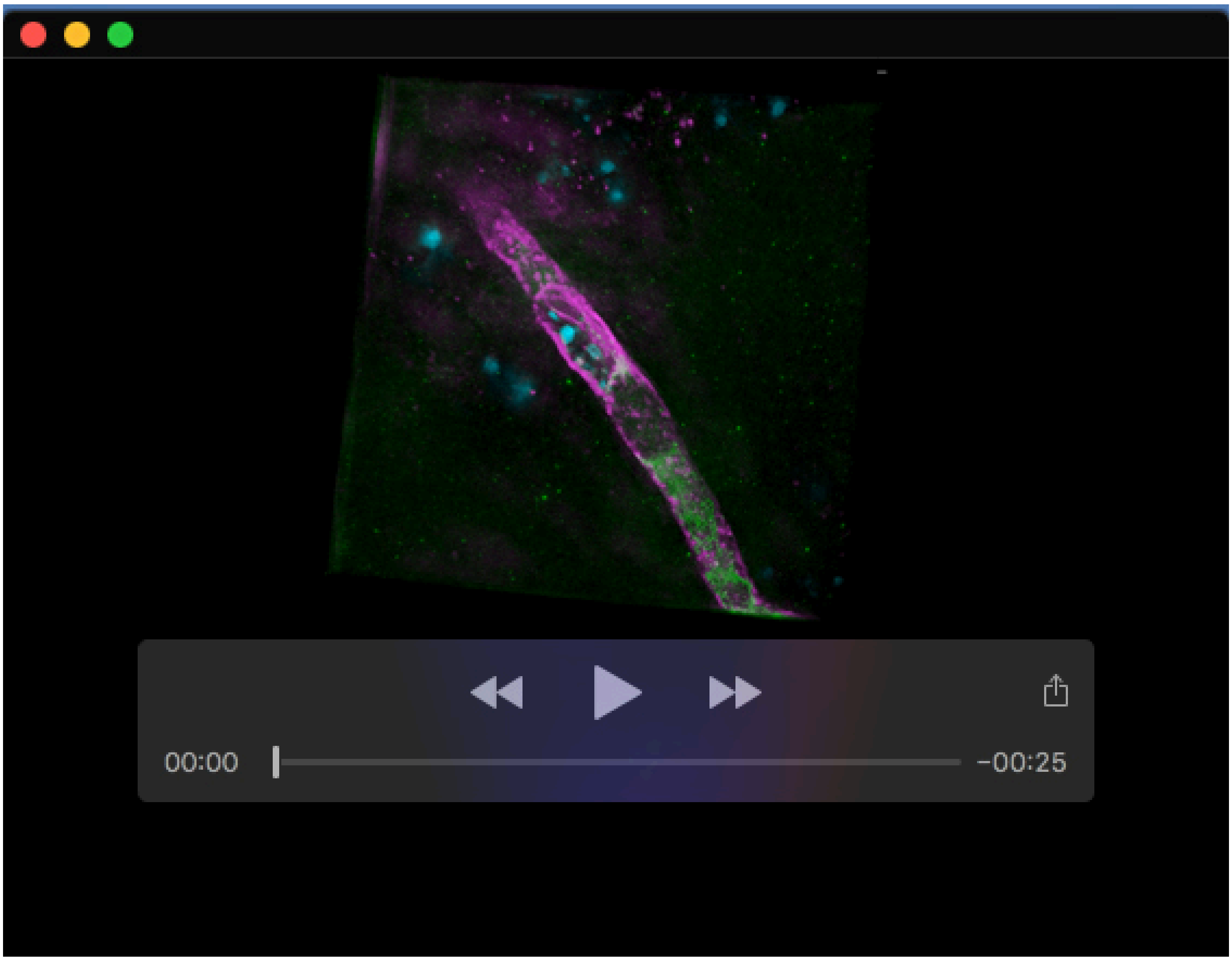

Movie 2. Super-resolution 3D structured illumination microscopy image showing absence of β-DG in the perivascular space in the cortex of *Dag1*$^{C667F/C667F}$ mice. Immunostaining for β-DG (green) in combination with lectin (magenta) to visualize blood vessels and Hoechst (cyan) in the cortex of 2-month-old *Dag1*$^{C667F/C667F}$ mice. 3D reconstruction of a 40.96 x 40.96 x 3 µm z-stack. This region of interest is also shown in Figure 5E.

Disease Models & Mechanisms • Supplementary information

Table S1. List of antibodies

Antibody (species)	Source	Identifier	Titer/Volume/ Dilution (when applicable)
α-Dystroglycan IIH6 (mouse)	Merck Millipore, Burlington, MA USA	RRID:AB_309828	IHC: 1:100<br>WB: 1:1000
α-Syntrophin (rabbit)	Alomone Labs, Jerusalem, Israel	RRID:AB_2756776	IHC: 1:300
Aquaporin 4 (rabbit)	Merck Millipore, Burlington, MA USA	RRID: AB_11210366	IHC: 1:1000<br>WB: 1:1000
BCL11A (mouse)	Abcam, Cambridge, UK	RRID: AB_2063996	IHC: 1:1000
β-Dystroglycan (mouse)	Novocastra, Newcastle upon Tyne, UK	RRID:AB_442043	IHC: 1:200
Calsequestrin (rabbit)	Thermo Fisher Scientific, Waltham, MA USA	RRID: AB_2071461	IHC: 1:500
CD31 (rabbit)	Abcam, Cambridge, UK	RRID: ab28364	IHC: 1:50
Dystroglycan (sheep)	R+D Systems, Minneapolis, MN USA	RRID: AB_10891298	IHC: 1:30<br>WB: 1000
Dystrophin (rabbit)	Abcam, Cambridge, UK	RRID: ab15277	IHC: 1:200
GFAP (chicken)	Merck Millipore, Burlington, MA USA	RRID: AB_177521	IHC: 1:500
GFAP (rabbit)	Dako, Santa Clara, CA, USA	RRID: AB_2811722	IHC: Neat
Glutamine Synthetase(rabbit)	Merck Millipore, Burlington, MA USA	RRID: AB_2110656	IHC: 1:500
GLT-1 (guinea pig)	Merck Millipore, Burlington, MA USA	RRID:AB_90949	IHC: 1:500
KIR4.1 (rabbit)	Alomone Labs, Jerusalem, Israel<br><br>Thermo Fisher, Waltham, MA USA	RRID: AB_2040120<br>RRID: PA5-37137	IHC: 1:200<br>WB: 1:500
NeuN (mouse)	Merck Millipore, Burlington, MA USA	RRID: AB_2298772	IHC: 1:500
pAKT (rabbit)	Fisher Scientific, Waltham, MA USA	RRID: 44623G	WB: 1_500
Pan-laminin (rabbit)	Abcam, Cambridge, UK	RRID:AB_298179	IHC: 1:500
PDGFRβ(mouse)	R&D Systems, Minneapolis, MN USA	RRID:AB_2162633	IHC: 1:50
S100β (rabbit)	Abcam, Cambridge, UK	RRID:AB_882426	IHC: 1:500
Tubulin-HRP (mouse)	Santa Cruz biotechnology, Dallas, TX US	RRID: Sc-23948	WB: 1:1000
Ubiquitin (rabbit)	Abcam, Cambridge, UK	RRID: AB134953	WB: 1:200
Alexa 488 donkey anti-mouse	Thermo Fisher Scientific, Waltham, MA USA	RRID:AB_141607	IHC: 1:500
Alexa 546 donkey	Thermo Fisher Scientific,	RRID:AB_2534016	IHC: 1:500

Disease Models & Mechanisms • Supplementary information

anti-rabbit	Waltham, MA USA		
Alexa 488 donkey anti-chicken	Jackson ImmunoResearch, Ely, Cambridgeshire, UK	RRID:AB_2313596	IHC: 1:500
Alexa 488 donkey anti-sheep	Thermo Fisher Scientific, Waltham, MA USA	RRID:AB_141362	IHC: 1:500
Fab mouse IgG (H&L) antibody goat polycolnal	Rockland Immunochemicals, Pennsylvania, PA USA	RRID: AB_218897	IHC blocking: 1:10
Alexa 594 Affinipure Fab fragment goat anti-mouse IgG (H+L)	Jackson ImmunoResearch, Ely, Cambridgeshire, UK	RRID: AB_2338900	IHC: 1:1000
Anti-rabbit-HRP	Advansta, USA		WB: 1:10000
Anti-sheep-HRP	R+D Systems, Minneapolis, MN USA		WB: 1:1000
